# A Multi-Species Analysis Defines Anaplerotic Enzymes and Amides as Metabolic Markers for Ammonium Nutrition

**DOI:** 10.3389/fpls.2020.632285

**Published:** 2021-01-27

**Authors:** María Begoña González-Moro, Itziar González-Moro, Marlon de la Peña, José María Estavillo, Pedro M. Aparicio-Tejo, Daniel Marino, Carmen González-Murua, Izargi Vega-Mas

**Affiliations:** ^1^Department of Plant Biology and Ecology, University of the Basque Country (UPV/EHU), Bilbao, Spain; ^2^Instituto Multidisciplinar de Biología Aplicada (IMAB), Universidad Pública de Navarra, Pamplona, Spain; ^3^Ikerbasque, Basque Foundation for Science, Bilbao, Spain

**Keywords:** ammonium, amino acid, glutamate dehydrogenase, glutamine synthease, isocitrate dehydrogenase, malic enzyme, phosphoenolpyruvate carboxylase

## Abstract

Nitrate and ammonium are the main nitrogen sources in agricultural soils. In the last decade, ammonium (NH_4_^+^), a double-sided metabolite, has attracted considerable attention by researchers. Its ubiquitous presence in plant metabolism and its metabolic energy economy for being assimilated contrast with its toxicity when present in high amounts in the external medium. Plant species can adopt different strategies to maintain NH_4_^+^ homeostasis, as the maximization of its compartmentalization and assimilation in organic compounds, primarily as amino acids and proteins. In the present study, we report an integrative metabolic response to ammonium nutrition of seven plant species, belonging to four different families: Gramineae (ryegrass, wheat, *Brachypodium distachyon*), Leguminosae (clover), Solanaceae (tomato), and Brassicaceae (oilseed rape, *Arabidopsis thaliana*). We use principal component analysis (PCA) and correlations among metabolic and biochemical data from 40 experimental conditions to understand the whole-plant response. The nature of main amino acids is analyzed among species, under the hypothesis that those Asn-accumulating species will show a better response to ammonium nutrition. Given the provision of carbon (C) skeletons is crucial for promotion of the nitrogen assimilation, the role of different anaplerotic enzymes is discussed in relation to ammonium nutrition at a whole-plant level. Among these enzymes, isocitrate dehydrogenase (ICDH) shows to be a good candidate to increase nitrogen assimilation in plants. Overall, metabolic adaptation of different carbon anaplerotic activities is linked with the preference to synthesize Asn or Gln in their organs. Lastly, glutamate dehydrogenase (GDH) reveals as an important enzyme to surpass C limitation during ammonium assimilation in roots, with a disparate collaboration of glutamine synthetase (GS).

## Introduction

Ammonium (NH_4_^+^) ion is a common metabolite in cells with an incongruous behavior. In plants, NH_4_^+^ can be taken up from the soil and incorporated into carbon (C) skeletons for the organic nitrogen (N) synthesis. At low concentrations in the nutrient solution or soil, NH_4_^+^ is typically the preferred N source for plants (Britto and Kronzucker, 2002), as its assimilation means a metabolic economy of eight electrons compared to nitrate (NO_3_^−^). Nevertheless, when NH_4_^+^ is accumulated in the cell above a certain threshold it causes toxicity, and most of the plants undergo visible symptoms such as reduced growth, increased root:shoot ratio, leaf chlorosis, as result of decreased net photosynthesis, reduced rhizosphere pH, and imbalance of mineral cations in plant tissues (Britto and Kronzucker, 2002; Szczerba et al., 2008). Similarly, as it happens in saline toxicity (Na^+^ stress), the competition between cation uptake and NH_4_^+^ influx, as well as the accumulation of positive charges in the cytosol, can break the membrane potential, compromising indirectly, for instance, the homeostasis of essential cations in the cell (Schulze et al., 2005; Szczerba et al., 2008). The breakdown of the ammonium homeostasis triggers multiple morphological and physiological responses in order for the cell to adapt to intracellular pH changes, osmotic potential, redox status, and metabolic processes (Patterson et al., 2010; Liu and von Wirén, 2017). Among the plant strategies to maintain ammonium homeostasis in the cell, two are highlighted: the maximization of the amount of NH_4_^+^ compartmentalized, presumably inside the vacuole, and the assimilation of NH_4_^+^ in organic compounds, primarily as amino acids and proteins. Both strategies are probably dependent on each other, as NH_4_^+^ would be accumulated in the cell once NH_4_^+^-assimilating capacity is exceeded by its uptake. Additionally, alternative N-containing compounds could be stored, as glucosinolates or glutathione (Coleto et al., 2017). A further strategy plants can follow is increasing NH_4_^+^ efflux to the external medium (Coskun et al., 2013). At whole-plant level, ammonium homeostasis can be mediated by its storage preferentially in roots (Setién et al., 2013; Vega-Mas et al., 2015) or be translocated to leaves (Sun et al., 2017), which points out to selective mechanisms of loading into the xylem and to different thresholds of ammonium sensitivity for root and leaf cells. Moreover, it is well-known that the ammonium toxicity thresholds are very disparate among species (Cruz et al., 2006; Esteban et al., 2016), and vary depending on environmental conditions as light intensity, atmospheric CO_2_ or pH (Setién et al., 2014; Vega-Mas et al., 2015; Sarasketa et al., 2016). In this line, the content of NH_4_^+^ in the cell is probably the stimulus that initiates cell responsive mechanisms. Indeed, above a certain threshold, NH_4_^+^ content could promote a scenario of stressing conditions for the cell. Therefore, internal NH_4_^+^ content could be hypothesized to act as primary metabolic marker of the stress degree, related with changes in C and N metabolism.

Ammonium (NH_4_^+^) is assimilated firstly by glutamine synthetase/glutamate synthase (GS/GOGAT) cycle (Lea and Miflin, 1974; Lea and Forde, 1994). Other accessory enzymatic pathways could also collaborate in the assimilation of NH_4_^+^, such as asparagine synthetase (AS) or glutamate dehydrogenase (GDH; Skopelitis et al., 2006; Gaufichon et al., 2016). In order to improve the synthesis of amino acids, carbon skeletons are demanded in the form of organic acids, which are provided by the tricarboxylic acid (TCA) cycle, that today is rather considered as an open cycle connected to other metabolic networks, such as anaplerotic routes (Tcherkez et al., 2009; Sweetlove et al., 2010). Indeed, the activities of some TCA and anaplerotic enzymes, as NADP-isocitrate dehydrogenase (ICDH), malic enzyme (ME), phosphoenolpyruvate carboxylase (PEPC), and malate dehydrogenase (MDH) have been reported to be regulated by ammonium nutrition (Setién et al., 2013; Coleto et al., 2017; de la Peña et al., 2019; Vega-Mas et al., 2019a). Thus, the flux of TCA intermediates would determine NH_4_^+^-assimilating capacity (Vega-Mas et al., 2019a). In this sense, environmental conditions that stimulate photosynthesis assimilation, and therefore the synthesis of carbohydrates, as high irradiance or CO_2_-enriched atmosphere have been shown to ameliorate the overall response of plants to ammonium stress (Setién et al., 2013; Vega-Mas et al., 2015). Accordingly, the accumulation of N-enriched compounds, primarily amino acids and proteins, could mirror the success of the metabolic capacity of the tissues to face up to ammonium nutrition, so they could be also considered as principal biomarkers of ammonium nutrition (Coleto et al., 2017; Vega-Mas et al., 2017). Amino acids and protein content overall respond positively to ammonium nutrition and both are determinant for the N-content of staple foods (Fuertes-Mendizábal et al., 2013; Hou et al., 2019). Major accumulated free amino acids vary on abundance depending on species although Glu, Gln, Asp, Asn, Ala, Pro, Ser, and Gly are generally the most abundant ones (Coleto et al., 2017; Vega-Mas et al., 2017; de la Peña et al., 2019). In particular, Asn and Gln deserve a special focus since they are primary assimilatory products (Vega-Mas et al., 2019a,b). Both are the main forms of N translocation within the plant, being highly abundant in xylem and phloem sap (Lea et al., 2007), and in the case of Gln, it plays a key role in the N status (Xu et al., 2012).

Despite the great knowledge gained about ammonium nutrition in the last decades, ammonium-resistance mechanisms are still poorly understood (Bittsánszky et al., 2015; Esteban et al., 2016; Liu and von Wirén, 2017). The comparison among plant species regarding their tolerance levels and physiological strategies to tackle ammonium nutrition is not easy for diverse reasons. Firstly, the experimentation has required tailoring the N concentrations in nutrient solutions to the optimal culture conditions, according to the growth systems employed for each plant species. Furthermore, studies have been done at different growth stages to reach the proper development of plants or even different environmental conditions. Secondly, apart from common markers as biomass accumulation and internal NH_4_^+^ content, no specific biomarkers for ammonium stress have been defined.

Focused on C and N metabolism, in the present study, we have assumed the challenge of integrating the response to ammonium nutrition of seven plant species, belonging to four different families: Gramineae (or Poaceae; ryegrass, wheat, *Brachypodium distachyon*), Leguminosae (or Fabaceae; clover), Solanaceae (tomato), and Brassicaceae (oilseed rape, *Arabidopsis thaliana*). These species include three crops (tomato, wheat, and oilseed rape), two grassland species (ryegrass and clover), and the model plants *Arabidopsis* and *Brachypodium*. Most of the analyzed data were originally published by our research group (Table 1; references herein), nevertheless, herein we provide new metabolic datasets for ryegrass, clover, and wheat. The aim of the present work is to integrate the metabolic response for the whole-plant systems and to establish common and unique traits involved in the metabolic ammonium homeostasis in several plants of agronomic interest in an attempt to select biomarkers indicative of ammonium nutrition or tolerance in plants. Moreover, we tested the hypothesis if the capacity of species to favor the Asn synthesis is related to their better performance under ammonium nutrition. The information gathered from this work could provide the basis for a better selection of plant varieties adapted to ammonium nutrition and to improve the handling of ammonium-based fertilizers.

**Table 1:** Growth conditions for plant species under different N regimes and environmental factors, plant age and organ studied.

Specie	Culture medium	Nitrogen source	N (mM)	Light (μmol m^−2^ s^−1^)	CO_2_ (ppm)	pH	N treatment (day)	Plant age at harvest (day)	Organ	References
**Ryegrass**										
*Lolium perenne* L.var. Herbus	liquid hydroponic	Ammonium Nitrate	0.5 2.5 5	300	400	6.5	21	35	Root Shoot	Belastegui-Macadam et al. (2007) 10.1016/j.jplph.2006.11.013
**Clover**										
*Trifolium repens* L.var. huia	liquid hydroponic	Ammonium Nitrate	0.5 2.5 5	300	400	6.5	21	42	Root Shoot	Belastegui-Macadam et al. (2007) 10.1016/j.jplph.2006.11.013
**Wheat**										
*Triticum aestivum* L.var. Cezanne	liquid hydroponic	Ammonium Nitrate	10	300 700	400	6.7	28	42	Root Shoot	Setién et al. (2013) 10.1016/j.jplph.2012.12.015
**Tomato**										
*Solanum lycopersicum* L.cv. Agora Hybrid F1	perlite: vermiculite (1:2, v:v)	Ammonium Nitrate	7.5 15	500	400 800	6.5	28	42	Root Leaves (3rd-4th)	Vega-Mas et al. (2015) 10.1016/j.plantsci.2015.09.021 Vega-Mas et al. (2017) 10.1093/pcp/pcx146
***Arabidopsis***										
*Arabidopsis thaliana* Col-0	liquid hydroponic (*in vitro*, sterile)	Ammonium Nitrate	2 10	200	400	5.7 6.7	12	21	Root Shoot	Sarasketa et al. (2016) 10.3389/fpls.2016.00029
**Oilseed rape**										
*Brassica napus cv.*Neptune	liquid hydroponic	Ammonium Nitrate	0.5 1	400	400	6.8–7.2	18	25	Root Leaves	Coleto et al. (2017) 10.1186/s12870-017-1,100-9
***Brachypodium***										
*Brachypodium distachyon*Bd21	liquid hydroponic	Ammonium Nitrate	1 2.5	350	400	6.8–7.2	24	35	Root Shoot	de la Peña et al. (2019) 10.1093/aobpla/plz029

## Materials and Methods

### Experimental Conditions

The crop and meadow plants selected for this study were ryegrass (*Lolium perenne* var. Herbus), clover (*Trifolium repens* L var. Huia), wheat (*Triticum aestivum* L. var. Cezanne), tomato (*Solanum lycopersicum* L cv. Agora Hybrid F1), *Arabidopsis* (*A. thaliana* Col-0), oilseed rape (*Brassica napus* cv. Neptune) and *Brachypodium* (*B. distachyon* Bd21). Table 1 compiles the total of 40 experimental growth conditions for both leaves and roots.

Plants were germinated and cultured under different environmental conditions (Table 1) in a phytotron (Servicio Fitotrón e Invernadero SGIKer, UPV/EHU) in a common 14/10 h light/darkness photoperiod with 22–24/18°C temperature regime and 60/70–80% relative humidity, respectively. Nitrogen source was ammonium or nitrate, applied as Ca(NO_3_)_2_ or (NH_4_)_2_SO_4_, respectively, at concentration from 0.5 to 15 mM depending on the plant species and culture medium. Light intensity (from 200–500 μmol photon m^−2^ s^−1^) was selected according to the physiological characteristics of the plant species. For wheat, 700 μmol photon m^−2^ s^−1^ were also supplied as high light intensity. Detailed germination and growth conditions, as well as precise composition of the nutrient solutions, are given for each species in the literature referenced in Table 1.

### Ammonium, Total Amino Acids, and Protein Quantification

Ammonium was determined from root and shoot extracts with the phenolhypochlorite-based colorimetric method or by ion-exchange chromatography in the case of ryegrass and clover (Dionex 600 equipment; see references in Table 1). For *Arabidopsis*, total free amino acids were quantified by the ninhydrin method (Yemm et al., 1955) as Sarasketa et al. (2014). For ryegrass and clover, amino acids were extracted as Belastegui-Macadam et al. (2007) and quantified by HPLC (JEOL Aminotac 500 JLC-500IV); and for wheat, tomato, *Brachypodium*, and oilseed by capillary electrophoresis (PA-800, Beckman Coulter Inc., United States; Setién et al., 2013).

Soluble protein was extracted from frozen tissues with similar extraction buffers as described in Setién et al. (2013) for wheat, Setién et al. (2014) for tomato, ryegrass, and clover, and in Sarasketa et al. (2016) for *Arabidopsis*, *Brachypodium*, and oilseed rape. Soluble protein content was determined by a dye binding protein assay (Bio-Rad Bradford Protein assay) using bovine serum albumin (BSA) as standard (Bradford, 1976).

### Nitrogen and Carbon Enzyme Activities

Enzyme activities were determined in a 96-well microplate reader (BioTek Instruments) from protein extracts. GS activity was measured monitoring the formation of γ-glutamilhydroxamate (γ-GHM) in a semibiosynthetic assay (Setién et al., 2013). GDH, PEPC, ICDH, and NAD-ME activities were measured monitoring the evolution of NAD(P)H monitored at 340 nm. GDH activity was determined in aminating sense (Setién et al., 2013). For ryegrass, wheat, and clover, novel data of carbon enzymes activities (PEPC, ICDH, and NAD-ME) were determined as previously described by Vega-Mas et al. (2015) or Sarasketa et al. (2016).

### Statistical Analysis

A total of 80 observations, given for root and leaf, are considered for the analyses comprising the seven species. The physiological and biochemical parameters were analyzed using FactoMineR software of R Commander (Le et al., 2008).^1^ A principal component analysis (PCA) was performed using the mean values of the measured parameters (Supplementary Table S1) along the different experiments according to Table 1. The mean value corresponds to several biological replicates (*n*) for each species, as follows: for ryegrass, clover, oilseed rape, and *Brachypodium n* = 4 (where each sample is a pool of ten plants); for wheat *n* = 3 (each sample being a pool of five plants); for tomato *n* = 9 or *n* = 3 (for amino acids); and for *Arabidopsis n* = 3 (where each sample is a pool of three plants). The data were visualized using PCA scores; this representation was used to show an overview of the entire dataset and to identify associations among the traits measured, as well as among the species. Pearson’s correlations were also run, and representations are shown when considered interesting.

## Results

### Physiological and Biochemical Markers to N Nutrition Are Organ-and Species-Dependent

In order to outline common physiological and metabolic traits in response to nitrogen nutrition a dataset of seven plant species has been considered in the present study. All species were grown with ammonium or nitrate as N source, although under different environmental factors that are known to affect ammonium tolerance, such as, medium pH, light intensity, or atmospheric CO_2_ concentration (Table 1). It must be noted that, at least, four different growth conditions were included for each species, and both organs, root and leaf, were considered in the study. Firstly, a whole-plant PCA was performed with the mean values corresponding to the physiological and metabolic parameters from both organs for the seven species, which included a total of 80 observations (Supplementary Table S1; Supplementary Figure S1). Nine parameters were considered including biomass, internal NH_4_^+^ content, total amino acid content (total AA), soluble protein content and enzymatic activities of GS, GDH, PEPC, NAD-ME, and ICDH. The principal components vectors, PC1 and PC2 explained 60.8% of the total variation (Supplementary Figure S1). In this whole-plant multi-species analysis, the first principal component (PC1) accounted for 41.9% of the total variance, without a clear separation of species along this axis. In contrast, PC2, which accounted for 18.9% of the variance, separated wheat from the rest of the species and came out to be more distant from *Arabidopsis*, suggesting clear differences in the response to nitrogen between these two species. Thus, GS activity and organ biomass were the markers that defined wheat response, while NAD-ME and GDH mostly defined the response of *Arabidopsis*.

**Figure 1:**
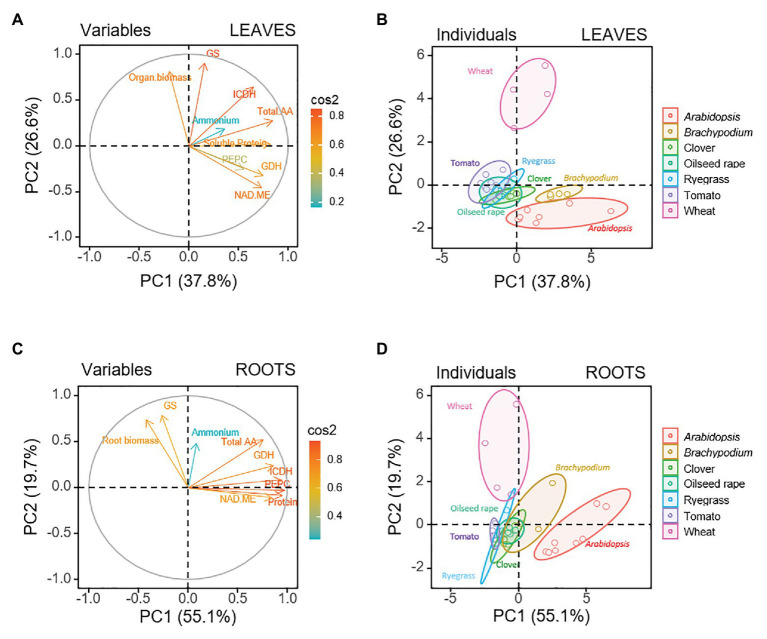
Multispecies principal component analysis (PCA) for leaf and root parts of seven plant species (*Arabidopsis*, *Brachypodium*, clover, oilseed rape, ryegrass, tomato, and wheat), considering physiological and biochemical variables: organ biomass, the content of ammonium, total amino acids and soluble protein, and the activity of GDH, GS, PEPC, NAD-ME, and ICDH. Loading plot of the physiological and biochemical variables for the first (PC1) and second (PC2) principal components for **(A)** leaves and **(C)** roots. Sample score of the individuals for **(B)** leaves and **(D)** roots.

Leaf and roots can show a different response to nitrogen nutrition, since ammonium is normally assimilated in roots meanwhile nitrate is preferentially assimilated in photosynthetic tissues (Yamaya and Kusano, 2014). Therefore, we considered necessary to carry out a second PCA analyzing separately data from roots (40 observations) and leaves (40 observations; Figure 1). Doing so, the variance explained by the first two PCs greatly increased. PC1 and PC2 together explained up to 74.8% (55.1% PC1 and 19.7% PC2; Figures 1C,D) for the root and to 64.4% (37.8% PC1 and 26.6% PC2; Figures 1A,B) for the leaf. Overall, as for the whole-plant multi-species analysis, the PCA for each organ clearly separated some species (Figures 1B,D). In the individuals representation for roots, only wheat and *Arabidopsis* remained distant to each other and clearly separated from the rest of species, that were situated in the middle and grouped closer among them (Figure 1D). This analysis indicated that GS and root biomass were driving factors in PC2 for wheat root, while soluble protein, PEPC, ICDH, and NAD-ME mainly contributed to *Arabidopsis* in PC1. For leaves soluble protein content, GDH and NAD-ME were the main positive markers of *Arabidopsis* and *Brachypodium* in the PC1 axis (Figures 1A,B), and both species placed separately from the intermediate group formed by tomato, ryegrass, clover, and oilseed rape. As in the root individuals plot, GS and biomass contributed as biomarkers for species segregation along PC2 in the leaf, being positive markers for wheat. Overall, leaf data showed a more clear separation than root data, which suggested that the leaf response to nitrogen nutrition was wider among species. Then, a subsequent PCA was performed for each species including variables related to N-compounds additional to the nine common variables previously considered. Among additional variables, N content, Asn, Gln, Glu, Asp, and Ser + Gly, as well as ratios of Gln/Glu, Asn/Asp, Asn/Gln, and Glu/Asp were included in the analysis for every species except for *Arabidopsis*, where only the former nine parameters were considered. The loading-plot when analyzing by PCA individually each species confirmed the separation between root and leaves for the seven species (Supplementary Figures S2, S3). Next, to extract information regarding specific responses to nitrogen nutrition we analyzed roots and leaves separately for each species.

### Root Species-Specific Physiological Traits to N Nutrition

**Figure 2:**
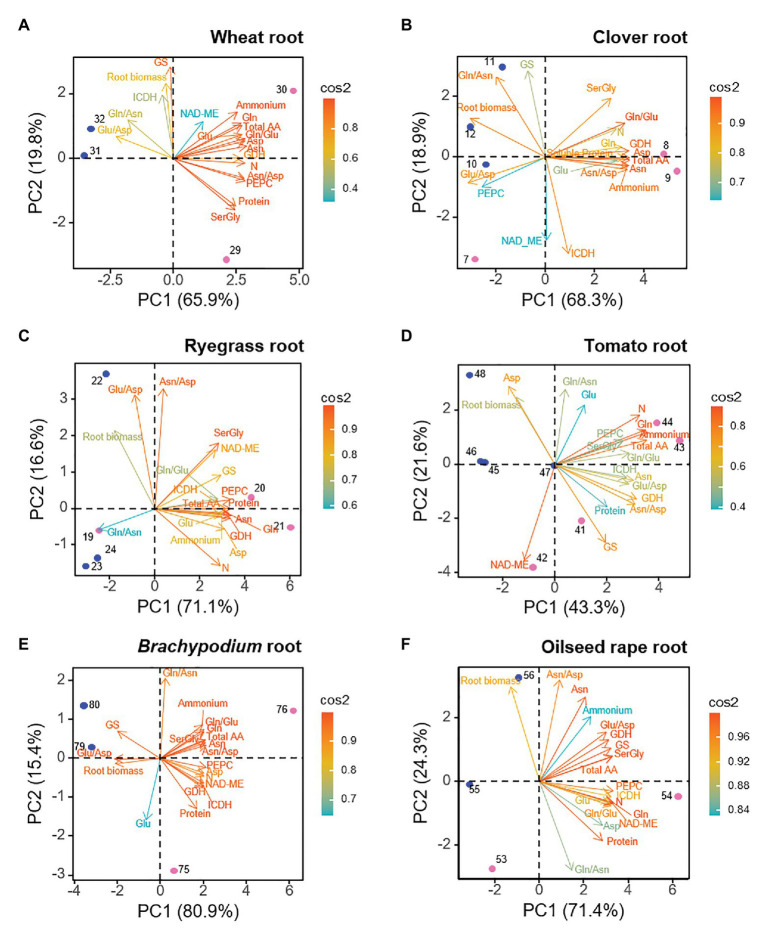
Loading plots from root dataset for each species, showing the relationships among the physiological and biochemical parameters. **(A)** wheat, **(B)** clover, **(C)** ryegrass, **(D)** tomato, **(E)** *Brachypodium*, and **(F)** oilseed rape. Points represent the sample score plot of the individuals for the first (PC1) and second (PC2) principal component, the number indicating the sample identity according to Supplementary Table S1. Blue and pink color stands for nitrate and ammonium nutrition, respectively.

The biplot PCAs for root data indicated that the two first PCs explained from a minimum of 64.9% to a maximum of 96.3% of the total variance (Figure 2). Observations of ammonium- and nitrate-fed root clearly separated along PC1 axis (43.3–80.9% of the variance). This revealed that the nitrogen nutrition imposed a different metabolic a biochemical functioning. In all the species several N-compounds (internal NH_4_^+^, total amino acids, Gln, Asn, Ser + Gly, and N content) contributed simultaneously to the metabolic profile of ammonium-fed roots, hence, we consider this group of traits as a root “N-cluster” responding to ammonium nutrition (Figure 2). Moreover, GDH behaved as a driving trait for ammonium-grown roots. Root biomass, in contrast, was a common positive marker for nitrate-fed roots; as well as ratios Glu/Asp or Gln/Asn for four out of six species, which can be taken as indicators of the preference of stored amine or amide in such species. GS activity correlated positively with nitrate-fed root of wheat, *Brachypodium*, but positively with ammonium-fed roots in the case of tomato and ryegrass. Overall, C-enzymes positioned closely to the “N-cluster” in ryegrass, *Brachypodium*, and oilseed rape, which suggested that C-enzymes would also be markers for ammonium nutrition. However, the general pattern showed a specific trend depending on each species. In the case of wheat, ICDH and NAD-ME segregated from “N-cluster” (Figure 2), as well as NAD-ME in *Arabidopsis* (Supplementary Figure S3). In tomato, Asp, Glu, and NAD-ME were separated from the main “N-cluster”. Besides, Asp was positioned closely to biomass, common positive marker for nitrate nutrition. And interestingly, clover was the unique species where PEPC acted as positive marker for nitrate-fed root. Correlations between the different parameters are shown in Supplementary Figure S4.

### Leaf Species- Specific Physiological Traits to N Nutrition

**Figure 3:**
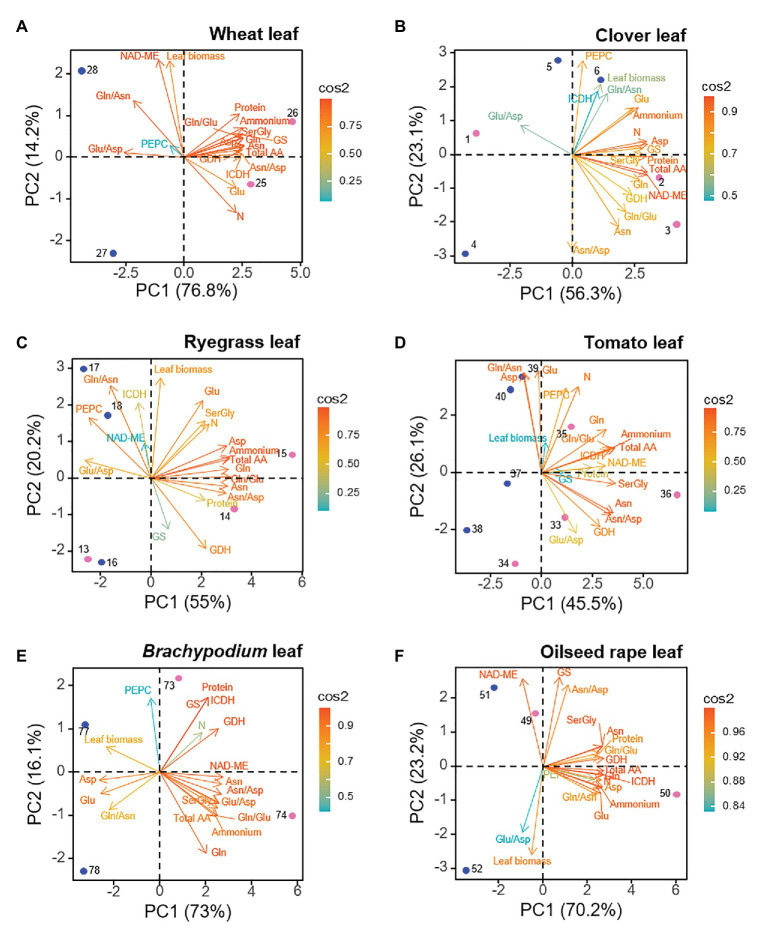
Loading plots from leaf dataset for each species, showing the relationships among the physiological and biochemical parameters. **(A)** wheat, **(B)** clover, **(C)** ryegrass, **(D)** tomato, **(E)** *Brachypodium*, and **(F)** oilseed rape. Points represent the sample score plot of the individuals for the first (PC1) and second (PC2) principal components, the number indicating the sample identity, according to Supplementary Table S1. Blue and pink color stands for nitrate and ammonium nutrition, respectively.

For leaf dataset, similarly to roots, the two first PCs explained a minimum of 73.5% and a maximum of 93.5% of the total variance depending on the species (Figure 3). Leaves corresponding to both N sources positioned segregated along PC1 axis, which explained between 45.5–76.8% of variance. As for roots, a “N-cluster” including main N-compounds (as internal NH_4_^+^, total amino acid, Gln and Asn, and soluble protein) could be defined as driver for ammonium nutrition in the leaf. Moreover, the variables in this leaf “N-cluster” showed overall a close correlation values with each other (Supplementary Figure S4). Interestingly, GDH was always closely related to the leaf “N-cluster” and GS also correlated positively to ammonium nutrition in wheat and clover leaves (Figure 3). On the other hand, leaf biomass was a clear positive marker for nitrate-fed leaves, in the case of *Brachypodium* and oilseed rape; a response also observed for *Arabidopsis* (Supplementary Figure S3). The behavior of leaf C-enzymes as biochemical marker for nitrogen nutrition was species-dependent, as their position in the PCA representation differed between species. For instance, although PCA did not revealed a clear separation between N nutrition in clover and ryegrass leaves, PEPC and ICDH were traits more positively correlated to nitrate-fed leaves. Nevertheless, for the rest of species, ICDH positively contributed as biochemical marker to leaves under ammonium nutrition (Figure 3; Supplementary Figure S3). In the case of NAD-ME, it was a positive marker for ammonium-fed leaves of clover, tomato and *Brachypodium*, but it behaved as a positive marker for nitrate-fed leaves of wheat, and oilseed rape.

## Discussion

The regulation of cell metabolism is crucial for the adaptation of plant growth and development to the changing environmental conditions. Indeed, the adequate metabolic management of the available N has great consequences for crop productivity. In this study, we have done an integration of main biochemical parameters in relation to C and N metabolism for seven species grown under exclusive ammonium or nitrate supply with the principal objective of looking for common and singular metabolic traits, indicatives of ammonium nutrition in crop plants.

The segregated distributions observed for the seven species when jointly analyzed at whole-plant level or organ-level by PCA (Supplementary Figure S1; Figure 1) illustrate the variety of plant performance regarding N nutrition. Particularly, the position of *Arabidopsis* and wheat, placed very distant from each other, would be indicative of their different biochemical adaptive response to the N source. The split of data corresponding to leaves and roots in the individual whole-plant score plot observed for the species (Supplementary Figure S2) would be in accordance with the dissimilar physiological role and underlying metabolism of both organs; and revealed the convenience of a separate analysis for each organ in response to the N nutrition. Furthermore, to deepen the insight regarding the potential specific biomarkers and relationships for ammonium nutrition in each organ, we carried out the PCA analysis for each species individually. In the same way, as for other chemical stresses in soils, as Na^+^, Cl^−^ or heavy metals (Chen et al., 2018), two strategies to cope with ammonium excess could be differentiated at whole-plant level. Those plants able to concentrate NH_4_^+^ at high levels in roots could be referred as “ammonium excluders” (or root-based mechanisms), as wheat, ryegrass, *Brachypodium*, clover, and oilseed rape; and those plants that keep similar NH_4_^+^ levels in both organs o even higher in the aerial parts could be considered as “ammonium includers” (or leaf-based mechanisms), as it seems the case for tomato and *Arabidopsis* (Supplementary Table S1; Supplementary Figure S5). In general, “excluder plants,” which follow an avoidance strategy keeping out damaging excess ions from translocation and uptake into shoots, are referred as tolerant species to ion stress (Tester and Davenport, 2003; Chen et al., 2018; Rossini-Oliva et al., 2018). Considering NH_4_^+^ is a key ion in N metabolism this classification must be taken with caution, since NH_4_^+^ accumulation in tissues results from the balance between NH_4_^+^ taken up by the roots plus NH_4_^+^ coming from catabolism but not (re)assimilated. In this sense, it is worth citing the exception of rice, considered an example of ammonium-tolerant crop that accumulates higher NH_4_^+^ levels in leaves (Sun et al., 2017), although under aerated conditions NH_4_^+^ assimilation occurs mainly in the roots (Xiaochuang et al., 2020). In the case of wheat, ryegrass, *Brachypodium*, and clover, but also in tomato, the internal NH_4_^+^ content in root could be considered as a good marker of ammonium nutrition, since this variable is one of the main drivers of PC1 (Figures 2, 3), probably because the capacity to incorporate NH_4_^+^ into amino acids is not saturated in these plants (Supplementary Figure S5). Only in the case of *Brachypodium* root and *Arabidopsis* in both organs, the capacity of NH_4_^+^ assimilation may be saturated as amino acid accumulation does not further increase for high NH_4_^+^ contents (Supplementary Figure S5). The measurement of free ammonium can be affected by non-specific interferences with other ammonium-compounds, such as amino acids Gly and Ala, when quantified by phenolhypochlorite-based assay (Ngo et al., 1982; Husted et al., 2000). However, as these amino acids were in minority in the analyzed tissues, such interferences would be minimal for the species included in the present work.

Several works have related stunted plant growth and biomass reduction as the main phenotypic markers of ammonium stress (Britto and Kronzucker, 2002; Sarasketa et al., 2014; Ijato et al., 2020). Accordingly to the results of PCAs and Pearson’s correlations, NH_4_^+^ accumulated in roots could have exceeded a certain tolerance threshold level in clover, *Arabidopsis* and *Brachypodium*, since growth of this organ was negatively affected (Figure 2; Supplementary Figures S3, S4). In contrast, globally, the leaf biomass would have been less affected by ammonium nutrition for the set of plant species studied, showing to be a negative marker only in the case of *Brachypodium* (Figure 3E). Therefore, these results seem to indicate that most of the species would have managed to display mechanisms at whole-plant level to maintain internal NH_4_^+^ levels in the photosynthetic organ in a relative non-toxic range.

### Total Free Amino Acid Content Is a Universal Marker Whereas Amides Are Specific Markers of Ammonium Tolerance

**Figure 4:**
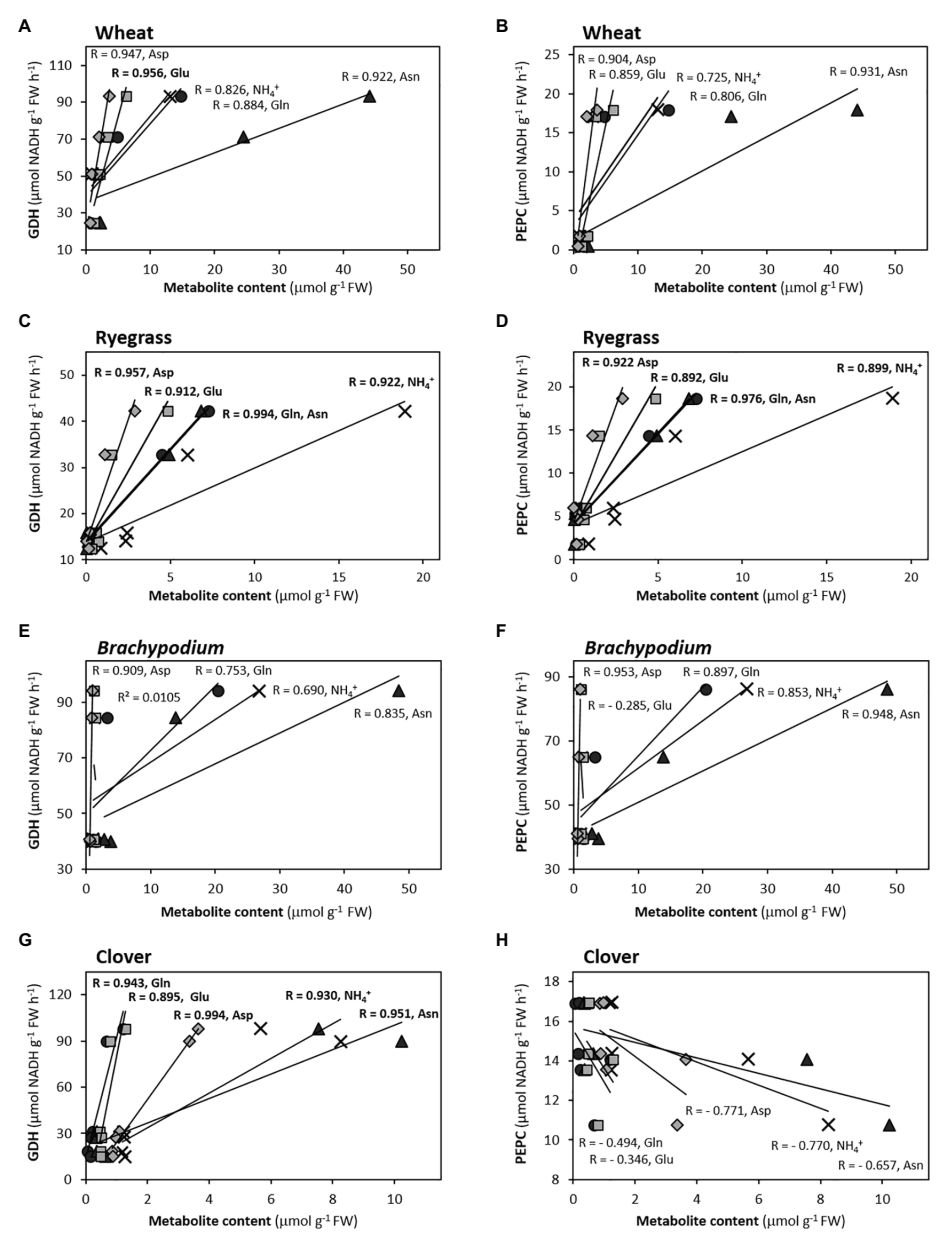
Correlations between GDH **(A,C,E,G)** and PEPC **(B,D,F,H)** with ammonium and main amino acids in roots of Asn-forming species, according to correlograms shown in Supplementary Figure S4. Pearson’s coefficient values are indicated (in bold, *p* < 0.05).

**Figure 5:**
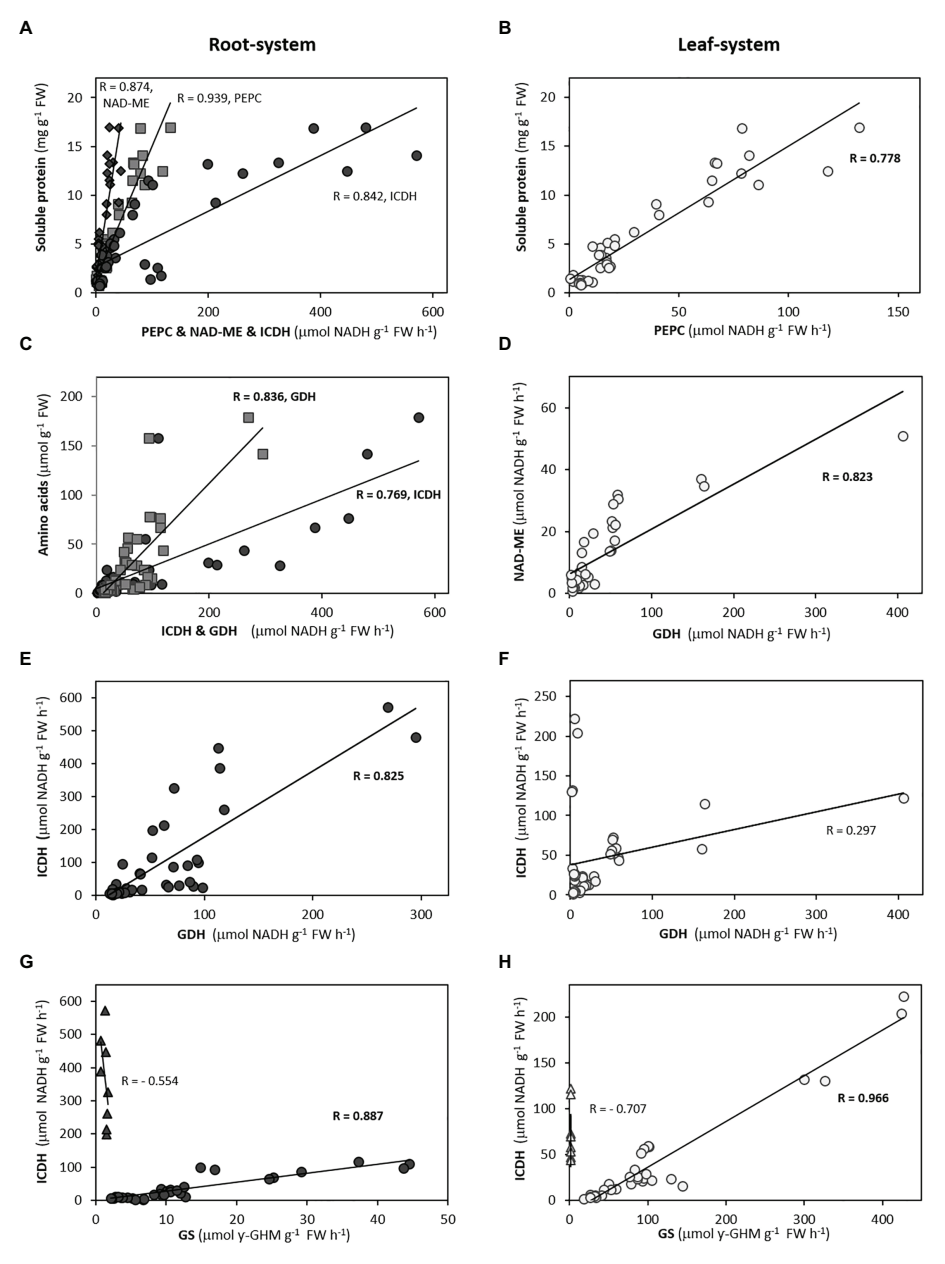
Selected correlations between biochemical and physiological variables in root-system **(A,C,E,G)** and leaf-system **(B,D,F,H)** for the ensemble of seven species. In the case of the correlation between GS and ICDH, *Arabidopsis* (triangles) is excluded for the general trend due to its low GS activity. Pearson’s coefficient values are indicated (in bold, *p* < 0.05).

Overall, the variation of total and soluble protein behaved as the principal contributors of PC1 in both organs (Figures 2, 3). Specially, the higher contribution of total amino acids to PC1 in *Brachypodium*, oilseed rape, and *Arabidopsis* roots (Figure 2; Supplementary Figure S3) would reflect their role as better biomarker of ammonium metabolism than the amount of NH_4_^+^ accumulated *per se*. Moreover, the higher correlation of total amino acid contents with soluble protein and C-enzymes (Supplementary Figure S4; Figures 4, 5C), comparing with NH_4_^+^, would also support their role as universal markers. Thus, the storage of NH_4_^+^ into amino acids and soluble protein could be considered as a metabolic detoxifying strategy, indicative of ammonium tolerance or the capacity to mitigate ammonium toxicity. The fundamental role of amino acid biosynthesis as principal detoxifying mechanism of NH_4_^+^ during plant response to ammonium nutrition remains valid for each species, as shown by the high slope of amino acid accumulation when plotted against NH_4_^+^ content in both organs (Supplementary Figure S5). Then, in general if NH_4_^+^ absorbed by the cell is not assimilated, its excess becomes toxic different ways for cells (Britto and Kronzucker, 2002) and reduces growth for most plant species. In this sense, the accumulation of inorganic N in form of NH_4_^+^ in tissues could be rather considered as an indicator of disturbance of NH_4_^+^ homeostasis (ion imbalance), which would trigger further cell or whole-plant responses (Sonoda et al., 2003; Ranathunge et al., 2014; Liu and von Wirén, 2017). Interestingly, considering the seven species together, the highly significant correlations of root amino acid content with some enzymes, as ICDH and GDH, but not with NH_4_^+^ content would indicate a possible role of ammonium-reduced compounds in the regulation of C and N-metabolism (Figures 4, 5; Supplementary Figure S4).

Among free amino acids, amides (Asn and Gln) perform an important role as N storage and long-distance N transporters thanks to their high N:C ratio, so they are reported as the primary N-reduced compounds that accumulate when plants are fed with NH_4_^+^ (Lea et al., 2007; Vega-Mas et al., 2019a,b). In the studied species, these amides represent, together with their respective precursors Glu and Asp, the major pool of amino acids, summing from 30 to 90% of total amino acid content (Supplementary Table S1). However, the nature of stored amides varies depending on species or families, since plants tend to accumulate either Asn or Gln, but also a mixed trend occurs. In monocots (wheat, ryegrass, and *Brachypodium*) and clover as well, the ammonium nutrition results in the accumulation of huge amounts of free Asn respect to nitrate nutrition (Supplementary Table S1); likewise accompanied by Gln in the case of ryegrass. The high contribution of Asn to PC1 in ammonium conditions would support its physiological role in ammonium nutrition (Figures 2, 3). Indeed, Asn is usually the main amino acid accumulated in some monocot species under ammonium nutrition (Setién et al., 2013; de Souza Miranda et al., 2016; de la Peña et al., 2019). Wheat, for instance, uses free Asn as N storage in the grain (Gao et al., 2016). In clover, the predominance of Asn under ammonium nutrition is in accordance with previous reports in temperate legumes such as pea or alfalfa (Pasqualini et al., 2001; Lea et al., 2007; Ariz et al., 2013). Interestingly, with the exception of *Brachypodium*, in Asn-accumulating species the equilibrium between amino acids vs. NH_4_^+^ accumulation was quite similar in root and leaf (similar slope; Supplementary Figure S5). This would indicate that the assimilatory capacity of inorganic N depends on NH_4_^+^ accumulated, which seems to occur in the same extent in photosynthetic and non-photosynthetic tissues within a given species. On the other side, in the case of tomato, Gln is the amino acid that keeps a better correlation with the internal NH_4_^+^ and with a high contribution to PC1, both for roots and leaves (Figures 2, 3; Supplementary Figure S4). Serine would also take importance in tomato metabolism, unlike monocots and clover. In oilseed rape roots, the separation of Asn from the principal cluster including reduced N-compounds (Figure 2) would match with the fact that Brassicaceae species are not specialists in accumulating N reduced in the form of Asn, probably because NH_4_^+^ is stored in the form of other amino acids, as Gln or Gly + Ser (Supplementary Table S1) or it can be derived to the synthesis of glucosinolates, N-compounds characteristic of this family (Marino et al., 2016; Coleto et al., 2017). Overall, we can propose that those species that tend to behave as “ammonium excluders” and show a tendency to detoxify NH_4_^+^ in the form of Asn are more tolerant to ammonium nutrition (wheat, *Brachypodium*, ryegrass, and clover). This proposal would agree with the fact that Asn is the most abundant amino acid in phloem and xylem of rice (Hou et al., 2019), a species considered as an ammonium-preferring crop (Zhu et al., 2009; Cao et al., 2018). Besides, Asn represents a 20% saving of carbon with respect to Gln and, as suggested initially by Ikeda et al. (2004), the asparagine synthase would be enhanced in Asn-accumulating species under ammonium nutrition similarly to its induction under abiotic stress (Lea et al., 2007).

### High Leaf GS Activity Contributes to Ammonium Homeostasis

Glutamine synthetase enzyme is the connecting point between inorganic NH_4_^+^ and organic N. Cytosolic GS (GS1) is responsible for primary NH_4_^+^ assimilation as well as reassimilation during germination or senescence, while chloroplastic GS (GS2) isoform is responsible for the assimilation of NH_4_^+^ coming from primary nitrate assimilation and photorespiration in leaves (Thomsen et al., 2014). Cytosolic GS1 is the predominant isoform in roots and its importance to confer tolerance when NH_4_^+^ is the sole N source for the plant has been reported (Guan et al., 2016; Sarasketa et al., 2016; Konishi et al., 2017). Interestingly, in wheat, the values for GS activity in both organs were very high and distant from other species, hampering the joint analysis of N nutrition response for the ensemble of the seven species (Supplementary Table S1; Figures 2, 3). Accordingly, to the high amino acid contents in roots (Supplementary Table S1; Supplementary Figure S5) we corroborate that NH_4_^+^ absorbed is primarily assimilated in this organ for the species studied, in the same line as several studies previously reported (Li et al., 2014; Liu and von Wirén, 2017). This means that root system manages to assimilate NH_4_^+^ despite having 4–10 times lower GS activities in comparison to photosynthetic organ in all the species. The exception was *Arabidopsis*, whose low GS activity showed similar values in both organs, and this could explain why the synthesis of amino acids seems to plateau in this species (Supplementary Figure S5). In general, there was neither a clear response of root GS activity to nitrogen nutrition nor a correlation with NH_4_^+^ or total amino acid content (Supplementary Figure S4). Overall, GS activity seems crucial to equip the leaf with high soluble protein or N, except for oilseed rape and *Arabidopsis* (Figure 3; Supplementary Figures S3, S4). Some authors have reported that GS is a major checkpoint for controlling the N status (Kichey et al., 2006), and plant species with higher GS activities seem more tolerant to ammonium nutrition (Cruz et al., 2006; Li et al., 2011). Among the several isogenes encoding GS, *GLN1;2* or *GS1;2* is essential for primary ammonium assimilation, mainly in roots, as revealed by the ammonium sensitivity displayed by mutants in this GS isoform (Yamaya and Kusano, 2014; Guan et al., 2016). The highest GS activity would be concordant with the fact that wheat, ryegrass, clover, and *Brachypodium* seemed to manage better the ammonium nutrition. In the case of wheat, *Brachypodium*, and clover, GS in leaf was positively correlated with internal NH_4_^+^ contents, along with the most of the variables grouped in the principal cluster (Figure 3; Supplementary Figure S4). Similar results were observed by Kichey et al. (2006) for wheat, with a strong relationship between shoot GS activity and total N content (NH_4_^+^ and amino acids), regardless the wheat cultivar examined or the N-feeding conditions. Nevertheless, recently, the ammonium toxicity has been attributed to an excessive assimilation of NH_4_^+^ in shoots by the plastidic GS (encoded by GLN2), which seems to promote an acidification in the cell, although the expression of root cytosolic GS (*GLN1.2*) ameliorated the ammonium toxicity (Hachiya et al., 2019). Since GS enzyme is subjected to a complex regulation (at transcriptional, post-transcriptional, and translational levels) with a negative allosteric behavior in the presence of some metabolites (Guan et al., 2016; Németh et al., 2018), and several mechanisms can contribute to regulate the cell pH in cells (Britto and Kronzucker, 2005), more studies are needed to clarify the role that this key enzyme exerts in plants ammonium tolerance, depending on the organ and the species. Future works will help to confirm if constitutive high GS activity, especially in roots, is a metabolic trait involved in ammonium tolerance.

### Glutamate Dehydrogenase as Universal Biomarker of Ammonium Nutrition in Roots

The duality of GDH functioning has been long-discussed. On one hand, functioning in its aminating sense may help preventing ammonium toxicity under stress conditions (Skopelitis et al., 2006) and works synthesizing glutamate during tomato fruit ripening (Ferraro et al., 2012) or during NH_4_^+^ reassimilation (Dubois et al., 2003; Grabowska et al., 2011). On the other hand, GDH may function in its deaminating sense for providing C skeletons, notably under C-limiting scenario (Glevarec et al., 2004; Grabowska et al., 2011; Fontaine et al., 2012). GDH activities are considerably higher in the root than in the leaf for the species included in this study, except *Arabidopsis* (Supplementary Table S1). Overall, the induction of GDH activity, especially in roots, has been previously reported as a response to ammonium nutrition (Lasa et al., 2002; Cruz et al., 2006; Setién et al., 2013; Sarasketa et al., 2016; de la Peña et al., 2019). In the present work, the positive response of GDH to NH_4_^+^, amino acids or Gln/Glu and Asn/Asp ratios (Supplementary Figure S4) for each species, would point out the involvement of this enzyme in the overall detoxifying of excess NH_4_^+^ into primary N-compounds. Interestingly, there is a positive correlation of GDH and ICDH with the total content of amino acids in the root-system for the ensemble of the seven species (Figure 5; Supplementary Figure S4). Thus, this high correlation could reflect 2-OG provision by ICDH at optimal rates to GDH for Glu synthesis, giving further support for a high Gln synthesis. Alternatively, GDH could be in charge of regulating the C/N ratio and function in the sense of Glu deamination to fuel back the 2-OG, in what can be considered as an anaplerotic entrance of C to the TCA. This deaminating role would make sense in the context of a severe ammonium toxicity whenever the release of NH_4_^+^ from Glu would be compensated with the further assimilation of 2 NH_4_^+^ molecules as a four-carbon skeletons Asn, thus saving carbon. Therefore, hypothetically the flexibility of GDH enzyme might lean the C provision toward Glu-Gln (if functioning as aminating) or Asp-Asn (if functioning as deaminating) synthesis.

In leaves, despite the inclusion of GDH, in general terms, in the main cluster of variables in the PCA (Figure 3), it shows a weaker response to internal NH_4_^+^, accordingly to the lower GDH activity values compared to the root (Supplementary Table S1; Supplementary Figure S4). The exception is *Arabidopsis*, where the GDH activity is considerably induced in leaves and maintains a high correlation with not only the total amino acid content but also with NH_4_^+^ content. Thus, high GDH activity could compensate the low leaf GS activity in this species considered as “ammonium includer,” and could explain that the total amino acids level in *Arabidopsis* leaves are in the same range than in the rest of species. It is also worth noting the apparently coordinated regulation of GDH and NAD-ME in the photosynthetic organ for the ensemble of seven species (Figure 5; Supplementary Figure S4). The use of GDH activity as an ammonium-tolerance marker really deserves to be considered in plants, regardless of the sense of the GDH reaction itself. Nevertheless, further experimentation is required for understanding the possible co-regulation of GDH with other TCA enzymes and to shed more light about the specificity toward its substrates *in vivo*.

### Anaplerotic Activities Responsible for the Enhanced C-Provision Necessary for Amino Acids Synthesis Are Coordinately Regulated

The derivation of NH_4_^+^ into specific amino acids and soluble proteins will depend not only on N-assimilating enzymes but also on C skeletons-providing enzymes. The common response of the dataset of the whole-plant showed that anaplerotic enzymes PEPC, NAD-ME, and ICDH were acting together to support the C skeletons provision for amino acids and soluble protein in response to N nutrition (Supplementary Figure S1). And the plasticity of these anaplerotic enzymes to match the C demand under different N regimes was further highlighted by the organ- and species-specific responses (Figures 2, 3) and correlations between variables (Figure 5; Supplementary Figure S4).

Phosphoenolpyruvate carboxylase has many roles in plant metabolism, one of its primary roles in roots being refixing the CO_2_ released during respiration to feed oxaloacetic acid (OAA) into the TCA cycle or to support the Asp+Asn formation. The high contribution of PEPC to PC1 in roots of Gramineae (wheat, ryegrass, and *Brachypodium*) and Brassicaceae (oilseed rape and *Arabidopsis*), close to the “N-cluster” (Figure 2), would note its role in the provision of C skeletons to avoid an overaccumulation of NH_4_^+^. The induction of PEPC under ammonium nutrition has been similarly described for other species (Lasa et al., 2002; Britto and Kronzucker, 2005; Ariz et al., 2013; Arias-Baldrich et al., 2017). However, PEPC in general is not well correlated with NH_4_^+^ content (Supplementary Figure S4), so this metabolite is not probably the factor that directly regulates PEPC activity. Indeed, our data suggest that downstream ammonium-derived products could be really upregulating PEPC, since in the root this activity keeps a strong correlation with amino acids, with Asn/Asp ratio in wheat and *Brachypodium*, with Gln and Asn in ryegrass, and with Gln in oilseed rape (R > 0.97; Supplementary Figure S4; Figure 4), main indicators of the N status in these plants. Indeed, Gln and Asn were also reported to upregulate PEPC at transcriptional level (Sugiharto et al., 1992; Suzuki et al., 1994). In this sense, the activation of PEPC in barley was dependent on the newly formed Gln, whenever GS is not inhibited (Díaz et al., 1995). Interestingly, despite the high Asn accumulation in the clover root, PEPC activity would not be participating as C anaplerotic enzyme in provision of OAA, if considering the negative correlation of PEPC with Asn (*R* = − 0.754; Supplementary Figure S4); as well as if considering its opposite position to the main cluster gathering the N-compounds in the PCA (Figure 2). This result in clover agrees with that obtained for alfalfa roots, where nitrate stimulated PEPC more effectively than ammonium (Pasqualini et al., 2001), but differs from the activation reported for this enzyme in pea roots upon ammonium nutrition (Lasa et al., 2002; Ariz et al., 2013). These contrasting responses suggest a more diverse regulation of anaplerotic PEPC in the provision of oxoacids within the Leguminosae family. Given the lack of PEPC responsiveness does not hamper the Asn formation in clover, alternative enzymes, as malate dehydrogenase, could be performing this role.

The presence of NAD-ME activity in mitochondria outfits the TCA cycle with an alternative pathway that allows the decarboxylation of malate rendering pyruvate that can be incorporated to TCA cycle (Wedding, 1989). Non-photosynthetic malic enzymes are key to govern malate-pyruvate balance, metabolites with multiple functions in the cell. Recently, the role of malic enzymes has also been reviewed in plant development and stress responses. For instance, malic enzymes can provide reductant power in the form of NAD(P)H, which are essential to remove reactive oxygen species under stressful scenarios (Sun et al., 2019). The proximate position of NAD-ME to the main “N-cluster” in roots, except for tomato and clover (Figure 2), suggests the coordinated regulation of this activity with other anaplerotic enzymes, PEPC and ICDH, to fuel the synthesis of oxoacids. Concerning mitochondrial isoform NAD-ME, a few papers described this enzyme as showing a positive response in the context of ammonium nutrition in spinach, pea, and tomato roots depending on the C-status of the plant (Kandlbinder et al., 1997; Lasa et al., 2002; Vega-Mas et al., 2015). Thus, the participation of NAD-ME seems to be claimed depending on the C-status of the plants, which is determined by the environmental growth conditions, as it occurs in tomato growing under elevated CO_2_, in such a way that under non-limiting C conditions the role of NAD-ME as anaplerotic enzyme may not be essential (Supplementary Table S1).

The enhanced TCA cycle, either by provision of OAA or pyruvate would ultimately promote the fuelling of C through ICDH activity to form 2-OG, the key organic acid regulating the coordination of C and N metabolism (Hodges et al., 2003). The cytosolic NADP-ICDH isoforms represent mostly (90–95%) the total ICDH activity (Chen, 1998; Sulpice et al., 2010; Leterrier et al., 2012). In ryegrass, tomato, *Brachypodium*, and oilseed rape, ICDH is well correlated with PC1, which leads to consider this enzyme as biomarker of ammonium nutrition, specifically in roots, and working in coordination with NAD-ME (Figure 3). Meanwhile, in wheat and clover, ICDH is more related to the PC2, but with dissimilar tendency in both species. In wheat, ICDH would support the high GS activity, while in clover it is opposed to GS and would be coordinately regulated with NAD-ME to provide oxoacids for amino acids synthesis (Figures 3, 5).

The role of photosynthetic organ to assimilate NH_4_^+^ excess was revealed more critical in those species behaving as “ammonium includers.” Overall, the storage of NH_4_^+^ into soluble protein in the leaf would be supported by PEPC response for the set of the seven species (Figure 5; Supplementary Figure S4). The positive relation of ICDH to the NH_4_^+^ accumulated and Asp or Glu formation (Supplementary Figure S4) in *Arabidopsis*, a species that can be considered among “ammonium includers”, and in oilseed rape, the weakest “ammonium excluder” among the species studied, would point it out as a key C-enzyme in an attempt to assimilate the NH_4_^+^ in the leaf in these species. The assimilation of NH_4_^+^ reaching photosynthetic tissues in “ammonium excluders” would be also favored by an enhanced ICDH, surely to endorse the high leaf GS activity characteristic of these species (Supplementary Table S1; Figure 5). With the exception of *Arabidopsis*, the fact that ICDH activity highly correlated with GS activity (Figure 5) in the multi-species representation would indicate this C enzyme is well-coordinated with N assimilation, probably to sustain the 2-OG production for the enhanced synthesis of Glu + Gln.

## Concluding Remarks

This multi-species study shows that NH_4_^+^ accumulation and the induction of its assimilatory mechanisms preferentially occur at root level and allow excluding NH_4_^+^ from the shoots. Monocot species (wheat, ryegrass, and *Brachypodium*), clover, and oilseed rape can be considered as “ammonium excluders.” Total content of amino acids, as primary nitrogen-reduced product, is shown as universal biomarker of ammonium metabolism in plant tissues, meanwhile internal NH_4_^+^ content would reflect an imbalance between the excessive NH_4_^+^ uptake and its assimilation capacity. The nature of main amino acids accumulated varies with the species, and the amide stored (Gln and/or Asn) behaves as a more specific biomarker of ammonium nutrition. Those Asn-accumulating species, as the case of monocots and clover, show a better response to ammonium nutrition. Besides, amino acid levels, particularly amides in roots, keep a strong correlation with the main enzymatic activities involved in NH_4_^+^ assimilation. Interestingly, the synthesis of amino acids in roots is not strictly dependent on the induction of GS activity, but it seems associated with a constitutive high GS activity. For this reason, root GS activity needs to be reconsidered as a species-dependent metabolic marker for ammonium nutrition. On the contrary, root GDH can be considered as a universal biomarker for ammonium nutrition, regardless of its specific aminating or deaminanting role during NH_4_^+^ assimilation. Furthermore, ICDH is revealed as an important enzyme to surpass C limitation during nitrogen assimilation. Overall, the species metabolic adaptation of different carbon anaplerotic activities was linked with the preference to synthesize Asn or Gln in their organs. Our findings provide several metabolic markers in the response of crop plants to ammonium nutrition, with interesting implications for the process of selecting crop plants with optimal response to ammonium nutrition.

## Data Availability Statement

The original contributions presented in the study are included in the article/Supplementary Material, further inquiries can be directed to the corresponding authors.

## Author Contributions

MBGM, IVM, and CGM: conceptualization. MBGM, MDLP, JME, PMAT, DM, and IVM: investigation and data collection. IGM, MBGM, and IVM: data analysis and writing of original draft. All authors contributed to the article and approved the submitted version.

### Conflict of Interest

The authors declare that the research was conducted in the absence of any commercial or financial relationships that could be construed as a potential conflict of interest.

## Footnotes

^1^http://factominer.free.fr/; http://www.jstatsoft.org/v25/i01/
