# Supplementary material for: A Multi-Species Analysis Defines Anaplerotic Enzymes and Amides as Metabolic Markers for Ammonium Nutrition
Source: Front Plant Sci. 2021 Jan 27;11:632285. doi: 10.3389/fpls.2020.632285 (PMC7873483; doi:10.3389/fpls.2020.632285)
Supplement: Supplementary file 2 [file Presentation_1.pdf]

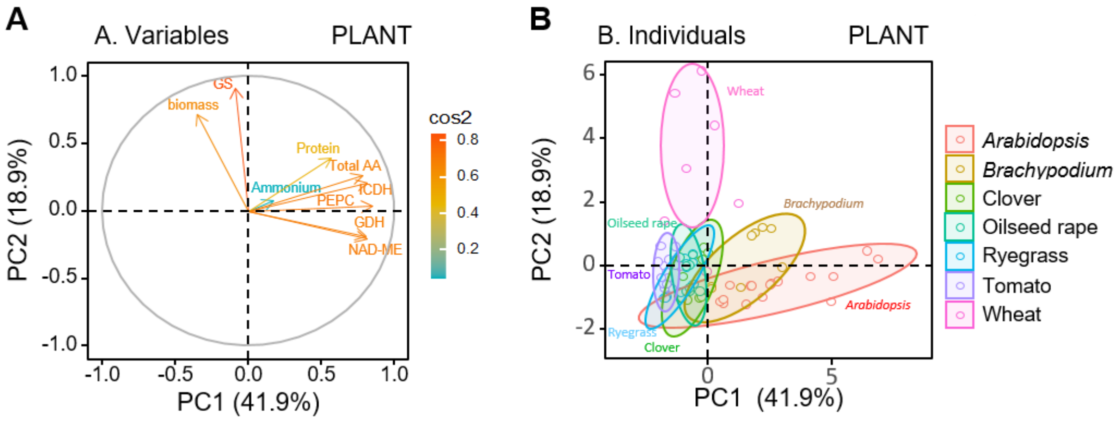

Figure S1. Whole-plant multispecies principal component analysis (PCA). (A) Loading plot of the common physiological and biochemical variables in root and leaf samples for 7 plant species: *Arabidopsis*, *Brachypodium*, clover, oilseed rape, ryegrass, tomato and wheat (legend on the right side). **(B)** Sample score plot of the individuals for the first (PC1) and second (PC2) principal components for the 7 plant species.

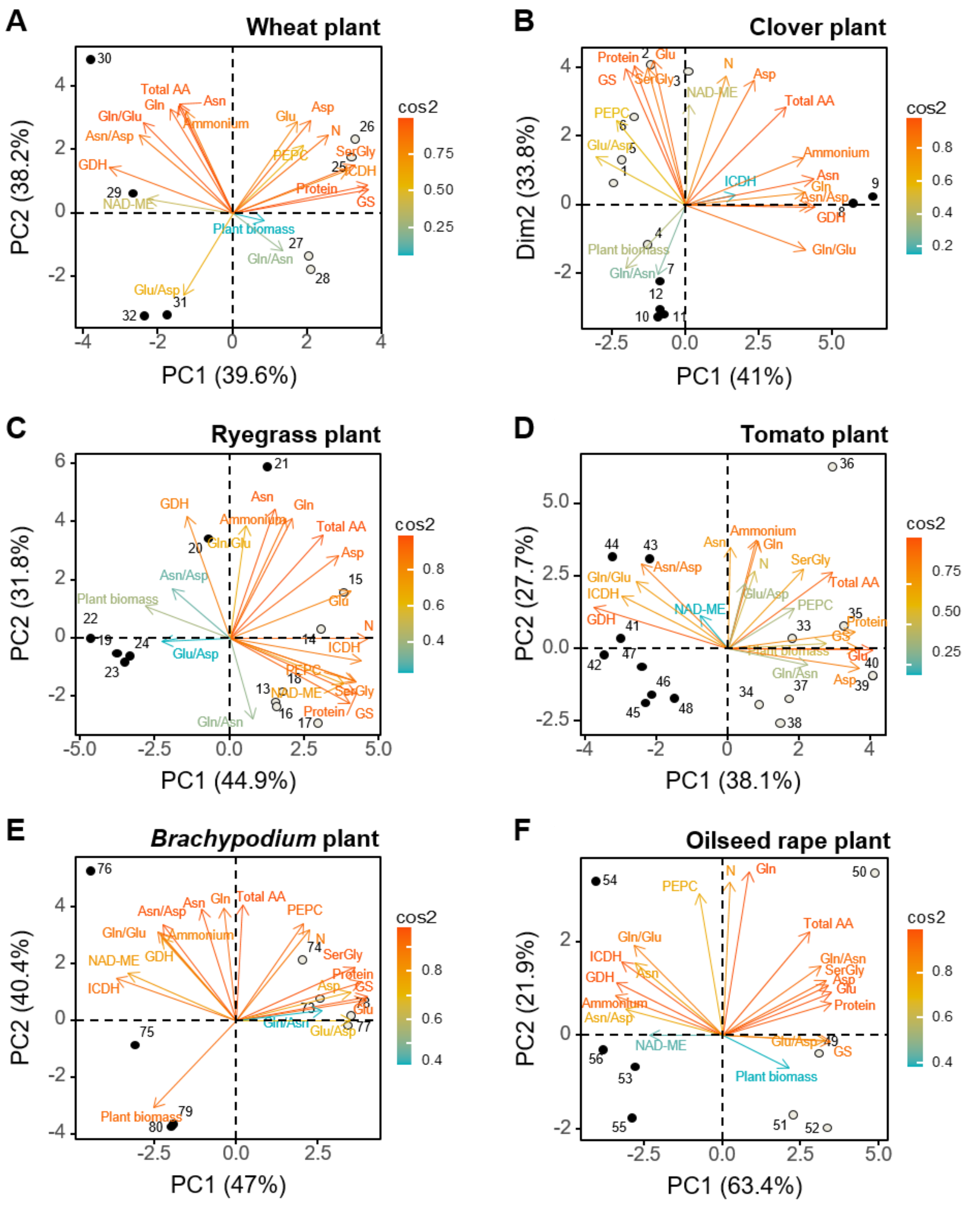

Figure S2. Loading plots from whole-plant dataset for each species, showing the relationships among the physiological and biochemical parameters. **(A)** wheat, **(B)** clover, **(C)** ryegrass, **(D)** tomato, **(E)** *Brachypodium* and **(F)** oilseed rape. Points represent the sample score plot of the individuals for the first (PC1) and second (PC2) principal component, the number indicating the sample identity according to Table S1. Black and white points stand for root, and leaf, respectively.

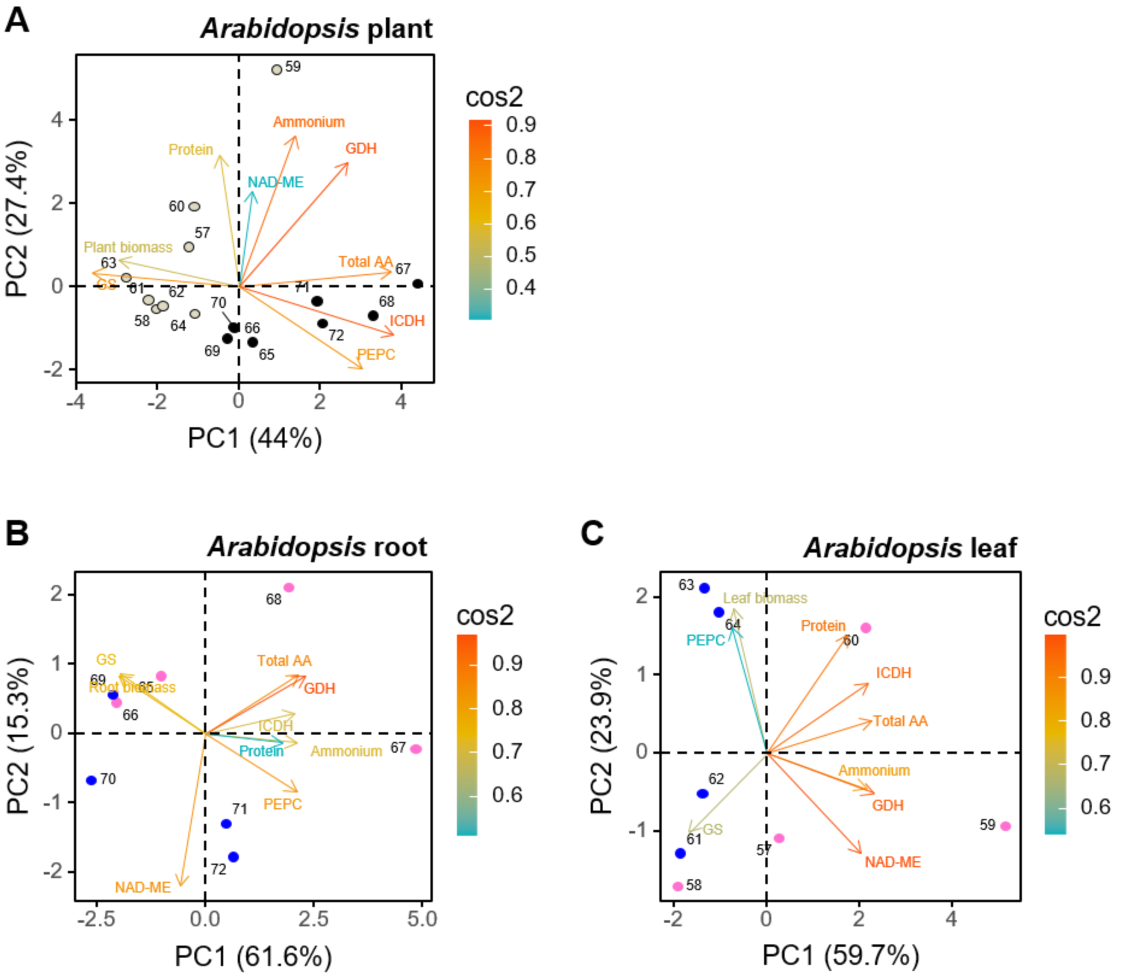

Figure S3. Loading plots from dataset for *Arabidopsis* (A-C), showing the relationships among the physiological and biochemical parameters. Points represent the sample score plot of the individuals for the first (PC1) and second (PC2) principal component, the number indicating the sample identity according to Table S1. Blue and pink points stand for nitrate and ammonium nutritions, respectively.

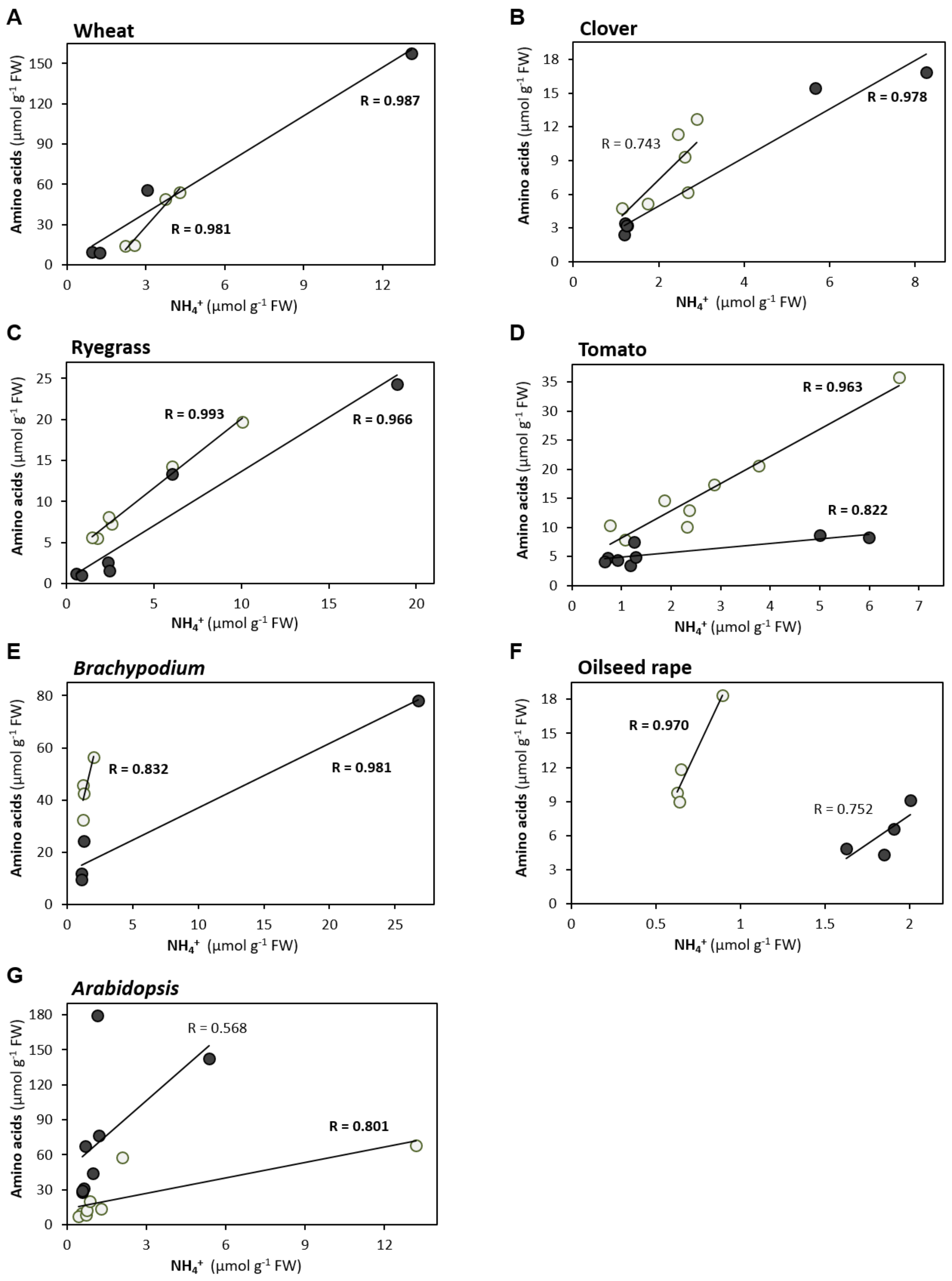

Figure S5. Correlations between total amino acid and NH_4^+ content in roots (black circle) and leaves (white circle) of different species. **(A)** wheat, **(B)** clover, **(C)** ryegrass, **(D)** tomato, **(E)** *Brachypodium*, **(F)** oilseed rape and **(G)** *Arabidopsis*. Pearson coefficient values are indicated (in bold, $p < 0.05$).
