# Supplementary material for: A Multi-Species Analysis Defines Anaplerotic Enzymes and Amides as Metabolic Markers for Ammonium Nutrition
Source: Front Plant Sci. 2021 Jan 27;11:632285. doi: 10.3389/fpls.2020.632285 (PMC7873483; doi:10.3389/fpls.2020.632285)
Supplement: Supplementary file 3 [file Presentation_2.pdf]

**Figure S4. Correlograms showing clustering according to the Pearson´s correlation coefficient on pairs between metabolites, enzymes activities and physiological parameters in roots and leaves of plants grown with ammonium and nitrate.** Significant positive and negative correlations ($p<0.05$) are highlighted in blue and red, respectively, according the colour scale shown on the right- or bottom side of the matrix. **(A)** wheat, **(B)** clover, **(C)** ryegrass, **(D)** tomato, **(E)** *Brachypodium* and **(F)** oilseed rape, **(G)** *Arabidopsis* and **(H)** ensemble of 7 plant species.

(A) Wheat:

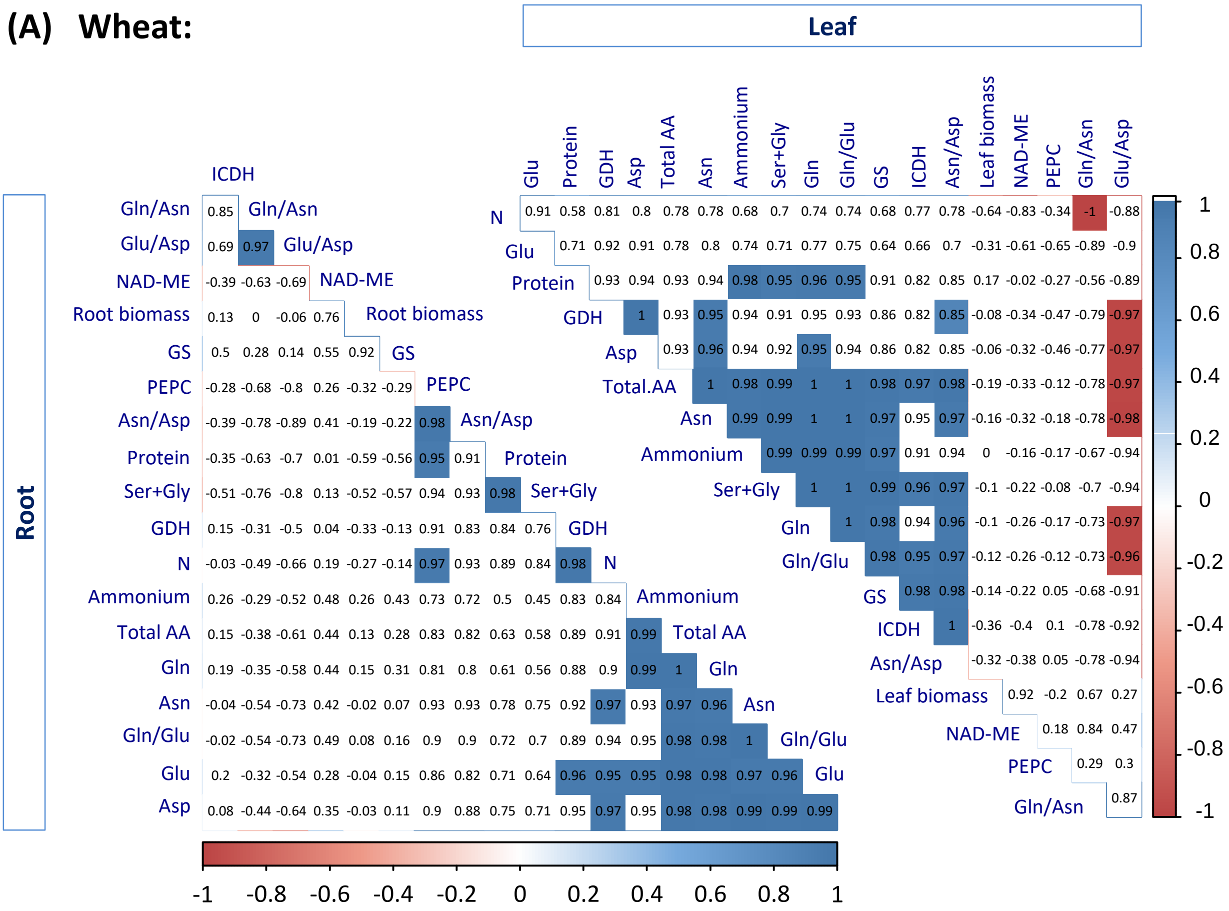

Leaf
Root
Glu
Protein
GDH
Asp
Total AA
Asn
Ammonium
Ser+Gly
Gln
Gln/Glu
GS
ICDH
Asn/Asp
Leaf biomass
NAD-ME
PEPC
Gln/Asn
Glu/Asp
N
Total.AA
Root biomass
1
0.8
0.6
0.4
0.2
0
-0.2
-0.4
-0.6
-0.8
-1

## (B) Clover:

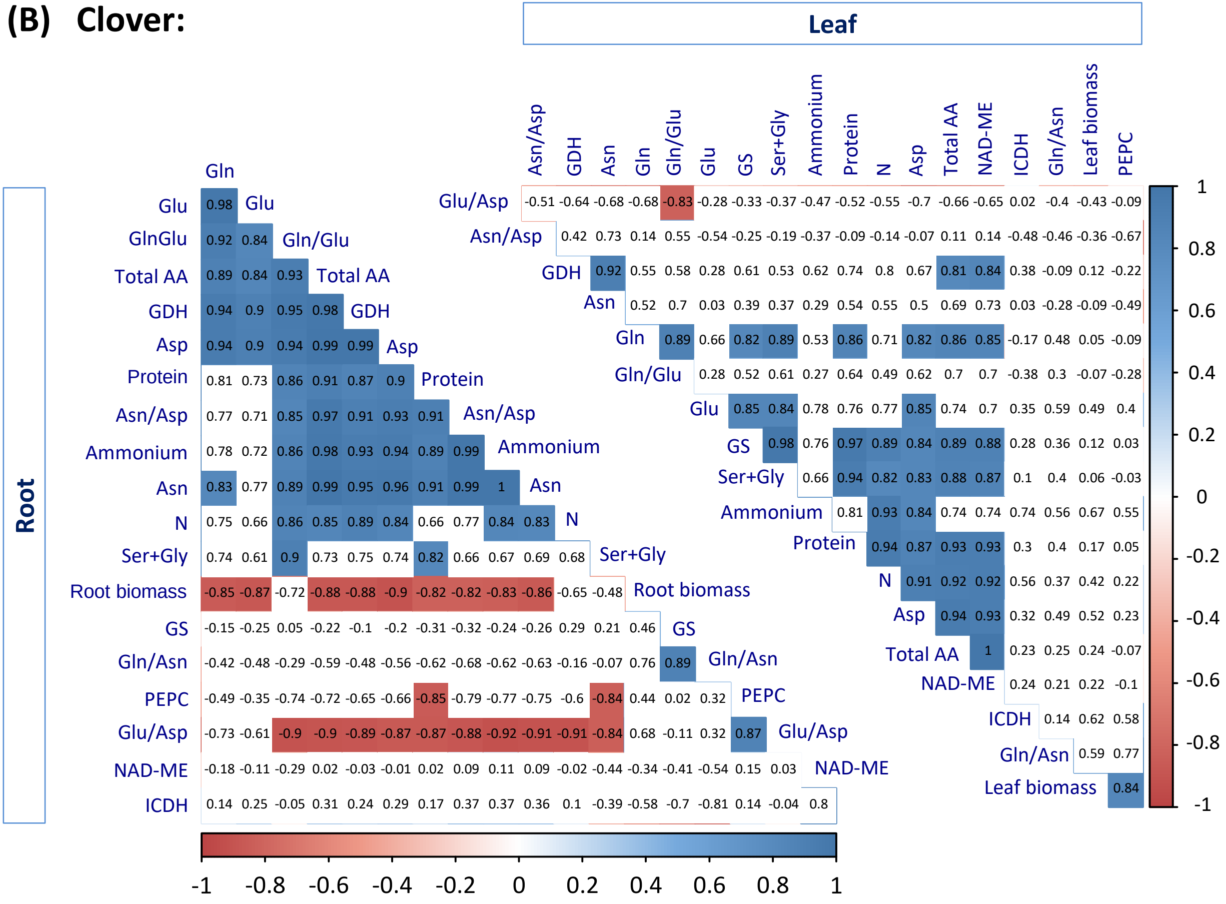
Leaf
Root
Gln
Glu 0.98 Glu
GlnGlu 0.92 0.84 Gln/Glu
Total AA 0.89 0.84 0.93 Total AA
GDH 0.94 0.9 0.95 0.98 GDH
Asp 0.94 0.9 0.94 0.99 0.99 Asp
Protein 0.81 0.73 0.86 0.91 0.87 0.9 Protein
Asn/Asp 0.77 0.71 0.85 0.97 0.91 0.93 0.91 Asn/Asp
Ammonium 0.78 0.72 0.86 0.98 0.93 0.94 0.89 0.99 Ammonium
Asn 0.83 0.77 0.89 0.99 0.95 0.96 0.91 0.99 1 Asn
N 0.75 0.66 0.86 0.85 0.89 0.84 0.66 0.77 0.84 0.83 N
Ser+Gly 0.74 0.61 0.9 0.73 0.75 0.74 0.82 0.66 0.67 0.69 0.68 Ser+Gly
Root biomass -0.85 -0.87 -0.72 -0.88 -0.88 -0.9 -0.82 -0.82 -0.83 -0.86 -0.65 -0.48 Root biomass
GS -0.15 -0.25 0.05 -0.22 -0.1 -0.2 -0.31 -0.32 -0.24 -0.26 0.29 0.21 0.46 GS
Gln/Asn -0.42 -0.48 -0.29 -0.59 -0.48 -0.56 -0.62 -0.68 -0.62 -0.63 -0.16 -0.07 0.76 0.89 Gln/Asn
PEPC -0.49 -0.35 -0.74 -0.72 -0.65 -0.66 -0.85 -0.79 -0.77 -0.75 -0.6 -0.84 0.44 0.02 0.32 PEPC
Glu/Asp -0.73 -0.61 -0.9 -0.9 -0.89 -0.87 -0.87 -0.88 -0.92 -0.91 -0.91 -0.84 0.68 -0.11 0.32 0.87 Glu/Asp
NAD-ME -0.18 -0.11 -0.29 0.02 -0.03 -0.01 0.02 0.09 0.11 0.09 -0.02 -0.44 -0.34 -0.41 -0.54 0.15 0.03 NAD-ME
ICDH 0.14 0.25 -0.05 0.31 0.24 0.29 0.17 0.37 0.37 0.36 0.1 -0.39 -0.58 -0.7 -0.81 0.14 -0.04 0.8
-1 -0.8 -0.6 -0.4 -0.2 0 0.2 0.4 0.6 0.8 1
Asn/Asp GDH Asn Gln Gln/Glu Glu GS Ser+Gly Ammonium Protein N Asp Total AA NAD-ME ICDH Gln/Asn Leaf biomass PEPC
Glu/Asp -0.51 -0.64 -0.68 -0.68 -0.83 -0.28 -0.33 -0.37 -0.47 -0.52 -0.55 -0.7 -0.66 -0.65 0.02 -0.4 -0.43 -0.09
Asn/Asp 0.42 0.73 0.14 0.55 -0.54 -0.25 -0.19 -0.37 -0.09 -0.14 -0.07 0.11 0.14 -0.48 -0.46 -0.36 -0.67
GDH 0.92 0.55 0.58 0.28 0.61 0.53 0.62 0.74 0.8 0.67 0.81 0.84 0.38 -0.09 0.12 -0.22
Asn 0.52 0.7 0.03 0.39 0.37 0.29 0.54 0.55 0.5 0.69 0.73 0.03 -0.28 -0.09 -0.49
Gln 0.89 0.66 0.82 0.89 0.53 0.86 0.71 0.82 0.86 0.85 -0.17 0.48 0.05 -0.09
Gln/Glu 0.28 0.52 0.61 0.27 0.64 0.49 0.62 0.7 0.7 -0.38 0.3 -0.07 -0.28
Glu 0.85 0.84 0.78 0.76 0.77 0.85 0.74 0.7 0.35 0.59 0.49 0.4
GS 0.98 0.76 0.97 0.89 0.84 0.89 0.88 0.28 0.36 0.12 0.03
Ser+Gly 0.66 0.94 0.82 0.83 0.88 0.87 0.1 0.4 0.06 -0.03
Ammonium 0.81 0.93 0.84 0.74 0.74 0.74 0.56 0.67 0.55
Protein 0.94 0.87 0.93 0.93 0.3 0.4 0.17 0.05
N 0.91 0.92 0.92 0.56 0.37 0.42 0.22
Asp 0.94 0.93 0.32 0.49 0.52 0.23
Total AA 1 0.23 0.25 0.24 -0.07
NAD-ME 0.24 0.21 0.22 -0.1
ICDH 0.14 0.62 0.58
Gln/Asn 0.59 0.77
Leaf biomass 0.84
1 0.8 0.6 0.4 0.2 0 -0.2 -0.4 -0.6 -0.8 -1

## (C) Ryegrass:

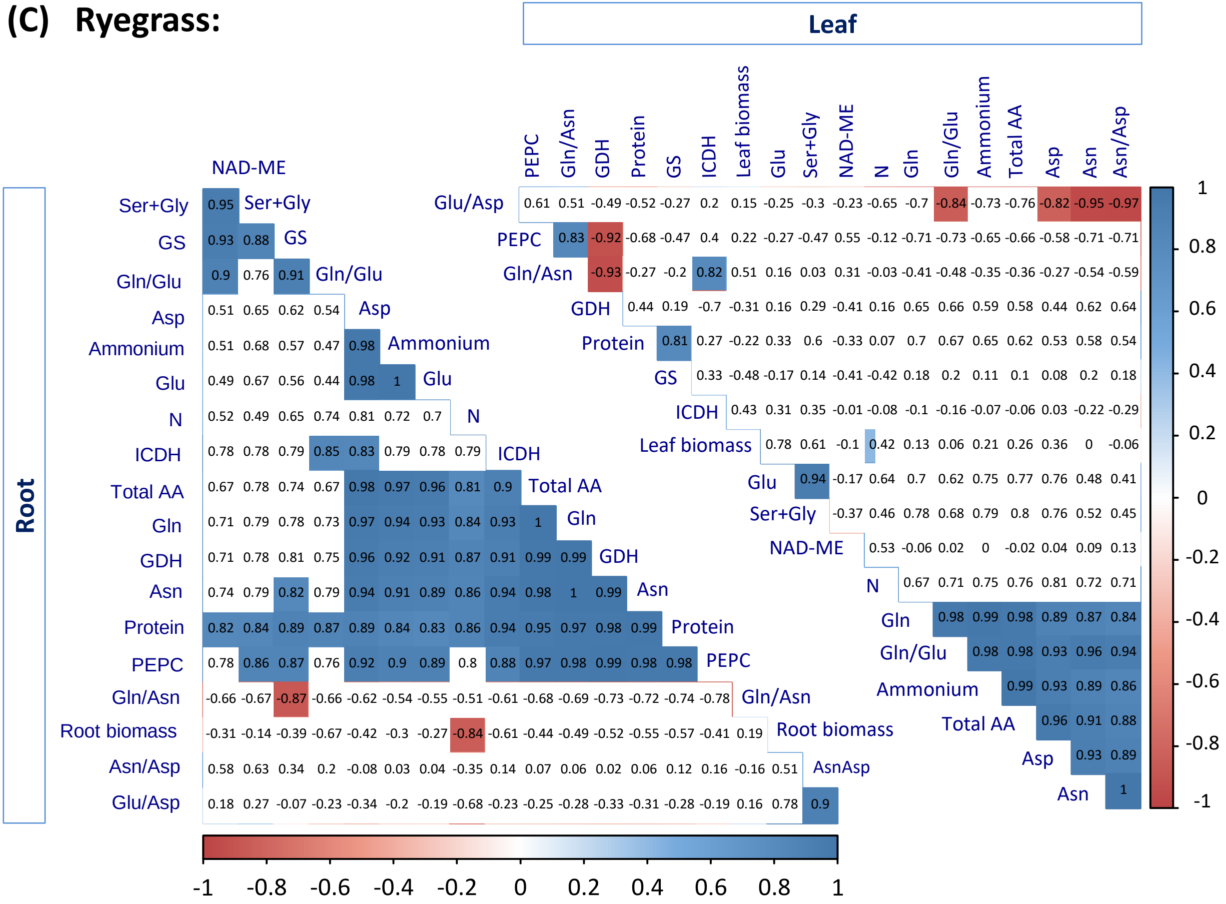
Leaf
PEPC Gln/Asn GDH Protein GS ICDH Leaf biomass Glu Ser+Gly NAD-ME N Gln Gln/Glu Ammonium Total AA Asp Asn Asn/Asp
Glu/Asp 0.61 0.51 -0.49 -0.52 -0.27 0.2 0.15 -0.25 -0.3 -0.23 -0.65 -0.7 -0.84 -0.73 -0.76 -0.82 -0.95 -0.97
PEPC 0.83 -0.92 -0.68 -0.47 0.4 0.22 -0.27 -0.47 0.55 -0.12 -0.71 -0.73 -0.65 -0.66 -0.58 -0.71 -0.71
Gln/Asn -0.93 -0.27 -0.2 0.82 0.51 0.16 0.03 0.31 -0.03 -0.41 -0.48 -0.35 -0.36 -0.27 -0.54 -0.59
GDH 0.44 0.19 -0.7 -0.31 0.16 0.29 -0.41 0.16 0.65 0.66 0.59 0.58 0.44 0.62 0.64
Protein 0.81 0.27 -0.22 0.33 0.6 -0.33 0.07 0.7 0.67 0.65 0.62 0.53 0.58 0.54
GS 0.33 -0.48 -0.17 0.14 -0.41 -0.42 0.18 0.2 0.11 0.1 0.08 0.2 0.18
ICDH 0.43 0.31 0.35 -0.01 -0.08 -0.1 -0.16 -0.07 -0.06 0.03 -0.22 -0.29
Leaf biomass 0.78 0.61 -0.1 0.42 0.13 0.06 0.21 0.26 0.36 0 -0.06
Glu 0.94 -0.17 0.64 0.7 0.62 0.75 0.77 0.76 0.48 0.41
Ser+Gly -0.37 0.46 0.78 0.68 0.79 0.8 0.76 0.52 0.45
NAD-ME 0.53 -0.06 0.02 0 -0.02 0.04 0.09 0.13
N 0.67 0.71 0.75 0.76 0.81 0.72 0.71
Gln 0.98 0.99 0.98 0.89 0.87 0.84
Gln/Glu 0.98 0.98 0.93 0.96 0.94
Ammonium 0.99 0.93 0.89 0.86
Total AA 0.96 0.91 0.88
Asp 0.93 0.89
Asn 1
1 0.8 0.6 0.4 0.2 0 -0.2 -0.4 -0.6 -0.8 -1
Root
NAD-ME
Ser+Gly 0.95 Ser+Gly
GS 0.93 0.88 GS
Gln/Glu 0.9 0.76 0.91 Gln/Glu
Asp 0.51 0.65 0.62 0.54 Asp
Ammonium 0.51 0.68 0.57 0.47 0.98 Ammonium
Glu 0.49 0.67 0.56 0.44 0.98 1 Glu
N 0.52 0.49 0.65 0.74 0.81 0.72 0.7 N
ICDH 0.78 0.78 0.79 0.85 0.83 0.79 0.78 0.79 ICDH
Total AA 0.67 0.78 0.74 0.67 0.98 0.97 0.96 0.81 0.9 Total AA
Gln 0.71 0.79 0.78 0.73 0.97 0.94 0.93 0.84 0.93 1 Gln
GDH 0.71 0.78 0.81 0.75 0.96 0.92 0.91 0.87 0.91 0.99 0.99 GDH
Asn 0.74 0.79 0.82 0.79 0.94 0.91 0.89 0.86 0.94 0.98 1 0.99 Asn
Protein 0.82 0.84 0.89 0.87 0.89 0.84 0.83 0.86 0.94 0.95 0.97 0.98 0.99 Protein
PEPC 0.78 0.86 0.87 0.76 0.92 0.9 0.89 0.8 0.88 0.97 0.98 0.99 0.98 0.98 PEPC
Gln/Asn -0.66 -0.67 -0.87 -0.66 -0.62 -0.54 -0.55 -0.51 -0.61 -0.68 -0.69 -0.73 -0.72 -0.74 -0.78 Gln/Asn
Root biomass -0.31 -0.14 -0.39 -0.67 -0.42 -0.3 -0.27 -0.84 -0.61 -0.44 -0.49 -0.52 -0.55 -0.57 -0.41 0.19 Root biomass
Asn/Asp 0.58 0.63 0.34 0.2 -0.08 0.03 0.04 -0.35 0.14 0.07 0.06 0.02 0.06 0.12 0.16 -0.16 0.51 AsnAsp
Glu/Asp 0.18 0.27 -0.07 -0.23 -0.34 -0.2 -0.19 -0.68 -0.23 -0.25 -0.28 -0.33 -0.31 -0.28 -0.19 0.16 0.78 0.9
-1 -0.8 -0.6 -0.4 -0.2 0 0.2 0.4 0.6 0.8 1

**(D) Tomato:**

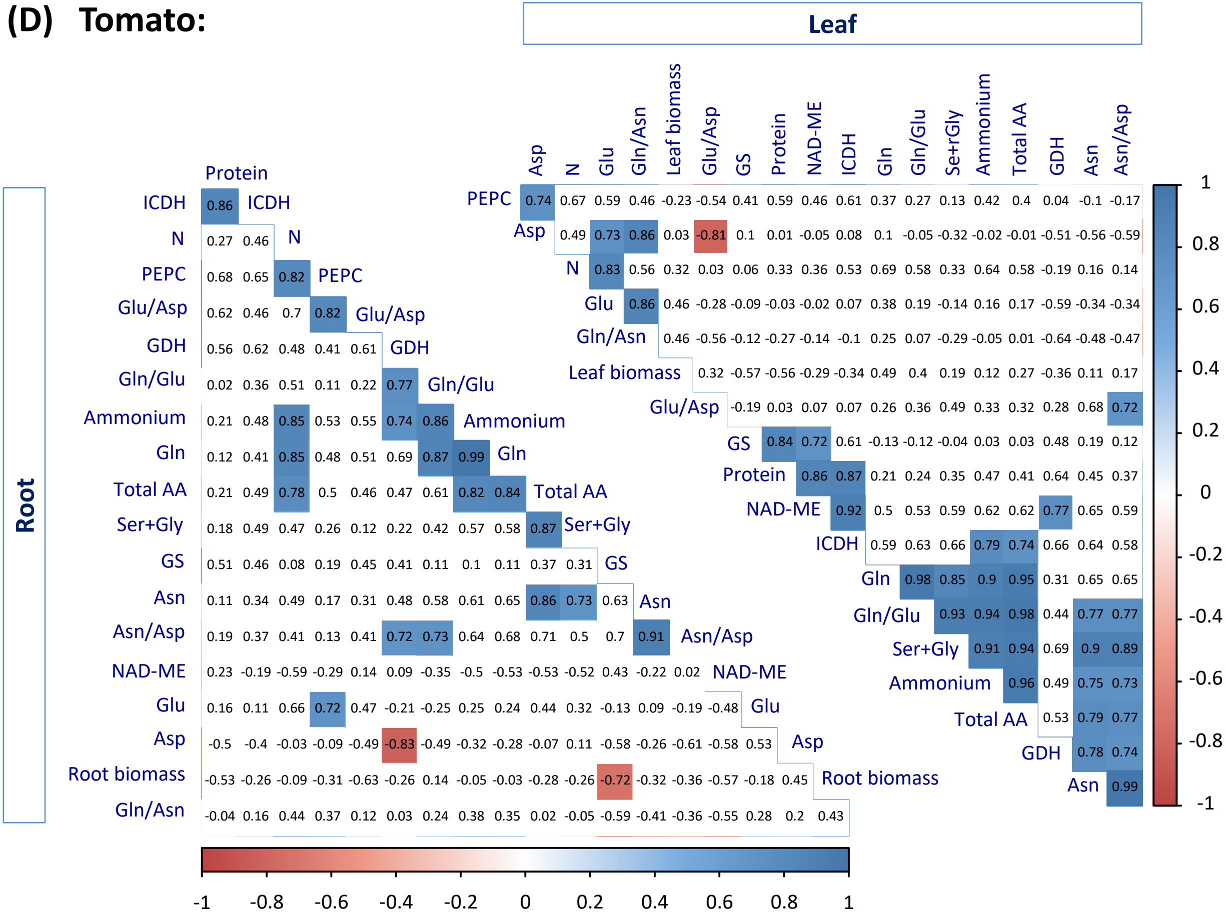
Leaf
Asp
N
Glu
Gln/Asn
Leaf biomass
Glu/Asp
GS
Protein
NAD-ME
ICDH
Gln
Gln/Glu
Se+rGly
Ammonium
Total AA
GDH
Asn
Asn/Asp
PEPC
Ser+Gly
Root
Protein
ICDH
N
PEPC
Glu/Asp
GDH
Gln/Glu
Ammonium
Gln
Total AA
Ser+Gly
GS
Asn
Asn/Asp
NAD-ME
Glu
Asp
Root biomass
Gln/Asn
1
0.8
0.6
0.4
0.2
0
-0.2
-0.4
-0.6
-0.8
-1

## (E) *Brachypodium*:

### Root

| | Glu | GS | Root biomass | Glu/Asp | Gln/Asn | Ammonium | Gln | Gln/Glu | Ser+Gly | Asn/Asp | Total AA | Asn | ICDH | GDH | NAD-ME | PEPC | N | Protein |
|---|---|---|---|---|---|---|---|---|---|---|---|---|---|---|---|---|---|---|
| GS | 0.14 | | | | | | | | | | | | | | | | | |
| Root biomass | 0.25 | 0.87 | | | | | | | | | | | | | | | | |
| Glu/Asp | 0.29 | 0.92 | 0.99 | | | | | | | | | | | | | | | |
| Gln/Asn | -0.61 | 0.26 | -0.2 | -0.12 | | | | | | | | | | | | | | |
| Ammonium | -0.55 | -0.71 | -0.93 | -0.92 | 0.5 | | | | | | | | | | | | | |
| Gln | -0.5 | -0.77 | -0.96 | -0.95 | 0.42 | 1 | | | | | | | | | | | | |
| Gln/Glu | -0.52 | -0.75 | -0.95 | -0.94 | 0.44 | 1 | 1 | | | | | | | | | | | |
| Ser+Gly | -0.4 | -0.81 | -0.99 | -0.97 | 0.34 | 0.98 | 0.99 | 0.99 | | | | | | | | | | |
| Asn/Asp | -0.43 | -0.86 | -0.98 | -0.99 | 0.26 | 0.97 | 0.99 | 0.98 | 0.99 | | | | | | | | | |
| Total AA | -0.44 | -0.83 | -0.98 | -0.98 | 0.32 | 0.98 | 0.99 | 0.99 | 1 | 1 | | | | | | | | |
| Asn | -0.43 | -0.85 | -0.98 | -0.98 | 0.29 | 0.97 | 0.99 | 0.99 | 0.99 | 1 | 1 | | | | | | | |
| ICDH | -0.07 | -0.99 | -0.9 | -0.94 | -0.24 | 0.72 | 0.78 | 0.76 | 0.83 | 0.87 | 0.84 | 0.86 | | | | | | |
| GDH | -0.1 | -1 | -0.86 | -0.92 | -0.28 | 0.69 | 0.75 | 0.74 | 0.8 | 0.85 | 0.82 | 0.84 | 1 | | | | | |
| NAD-ME | -0.18 | -1 | -0.87 | -0.93 | -0.23 | 0.72 | 0.78 | 0.77 | 0.82 | 0.88 | 0.84 | 0.86 | 0.99 | 1 | | | | |
| PEPC | -0.29 | -0.97 | -0.95 | -0.98 | -0.02 | 0.85 | 0.9 | 0.89 | 0.92 | 0.96 | 0.94 | 0.95 | 0.97 | 0.97 | 0.98 | | | |
| N | -0.29 | -0.99 | -0.87 | -0.93 | -0.16 | 0.76 | 0.81 | 0.8 | 0.83 | 0.89 | 0.86 | 0.88 | 0.97 | 0.98 | 0.99 | 0.98 | | |
| Protein | 0.27 | -0.91 | -0.75 | -0.79 | -0.48 | 0.47 | 0.55 | 0.53 | 0.64 | 0.68 | 0.64 | 0.66 | 0.94 | 0.93 | 0.9 | 0.83 | 0.84 | |
| Asp | -0.01 | -0.9 | -0.97 | -0.96 | -0.02 | 0.81 | 0.85 | 0.84 | 0.91 | 0.91 | 0.9 | 0.9 | 0.94 | 0.91 | 0.9 | 0.94 | 0.87 | 0.88 |

Color scale: -1, -0.8, -0.6, -0.4, -0.2, 0, 0.2, 0.4, 0.6, 0.8, 1

### Leaf

| | Asn | Total AA | Ser+Gly | Glu/Asp | Ammonium | Gln/Glu | NAD-ME | Asn/Asp | N | GDH | GS | Protein | ICDH | PEPC | Gln/Asn | Glu | Leaf biomass | Asp |
|---|---|---|---|---|---|---|---|---|---|---|---|---|---|---|---|---|---|---|
| Gln | 0.77 | 0.86 | 0.85 | 0.86 | 0.91 | 0.92 | 0.75 | 0.84 | 0.25 | 0.42 | 0.13 | 0.13 | 0.13 | -0.53 | -0.37 | -0.78 | -0.57 | -0.66 |
| Asn | | 0.97 | 0.98 | 0.88 | 0.84 | 0.88 | 0.96 | 0.95 | 0.75 | 0.88 | 0.7 | 0.67 | 0.66 | -0.37 | -0.86 | -0.75 | -0.83 | -0.87 |
| Total AA | | | 1 | 0.85 | 0.83 | 0.88 | 0.9 | 0.91 | 0.7 | 0.75 | 0.54 | 0.49 | 0.48 | -0.55 | -0.79 | -0.7 | -0.71 | -0.77 |
| Ser+Gly | | | | 0.86 | 0.84 | 0.88 | 0.92 | 0.92 | 0.71 | 0.78 | 0.57 | 0.52 | 0.51 | -0.52 | -0.81 | -0.72 | -0.74 | -0.8 |
| Glu/Asp | | | | | 0.99 | 0.99 | 0.95 | 0.98 | 0.37 | 0.75 | 0.53 | 0.57 | 0.58 | -0.11 | -0.56 | -0.97 | -0.9 | -0.95 |
| Ammonium | | | | | | 1 | 0.91 | 0.96 | 0.27 | 0.66 | 0.41 | 0.46 | 0.47 | -0.16 | -0.46 | -0.97 | -0.84 | -0.9 |
| Gln/Glu | | | | | | | 0.93 | 0.98 | 0.35 | 0.69 | 0.45 | 0.48 | 0.48 | -0.22 | -0.53 | -0.95 | -0.84 | -0.9 |
| NAD-ME | | | | | | | | 0.99 | 0.62 | 0.91 | 0.74 | 0.75 | 0.75 | -0.13 | -0.77 | -0.89 | -0.95 | -0.97 |
| Asn/Asp | | | | | | | | | 0.52 | 0.83 | 0.63 | 0.64 | 0.64 | -0.19 | -0.69 | -0.92 | -0.91 | -0.95 |
| N | | | | | | | | | | 0.78 | 0.79 | 0.68 | 0.66 | -0.4 | -0.97 | -0.18 | -0.49 | -0.48 |
| GDH | | | | | | | | | | | 0.95 | 0.94 | 0.94 | 0.04 | -0.9 | -0.68 | -0.92 | -0.9 |
| GS | | | | | | | | | | | | 0.99 | 0.98 | 0.21 | -0.87 | -0.47 | -0.81 | -0.75 |
| Protein | | | | | | | | | | | | | 1 | 0.33 | -0.79 | -0.55 | -0.86 | -0.8 |
| ICDH | | | | | | | | | | | | | | 0.36 | -0.77 | -0.56 | -0.86 | -0.81 |
| PEPC | | | | | | | | | | | | | | | 0.3 | -0.09 | -0.19 | -0.1 |
| Gln/Asn | | | | | | | | | | | | | | | | 0.4 | 0.68 | 0.67 |
| Leaf biomass | | | | | | | | | | | | | | | | | 0.9 | 0.93 |
| Glu | | | | | | | | | | | | | | | | | | 0.99 |

Color scale: 1, 0.8, 0.6, 0.4, 0.2, 0, -0.2, -0.4, -0.6, -0.8, -1

# (F) Oilseed rape:

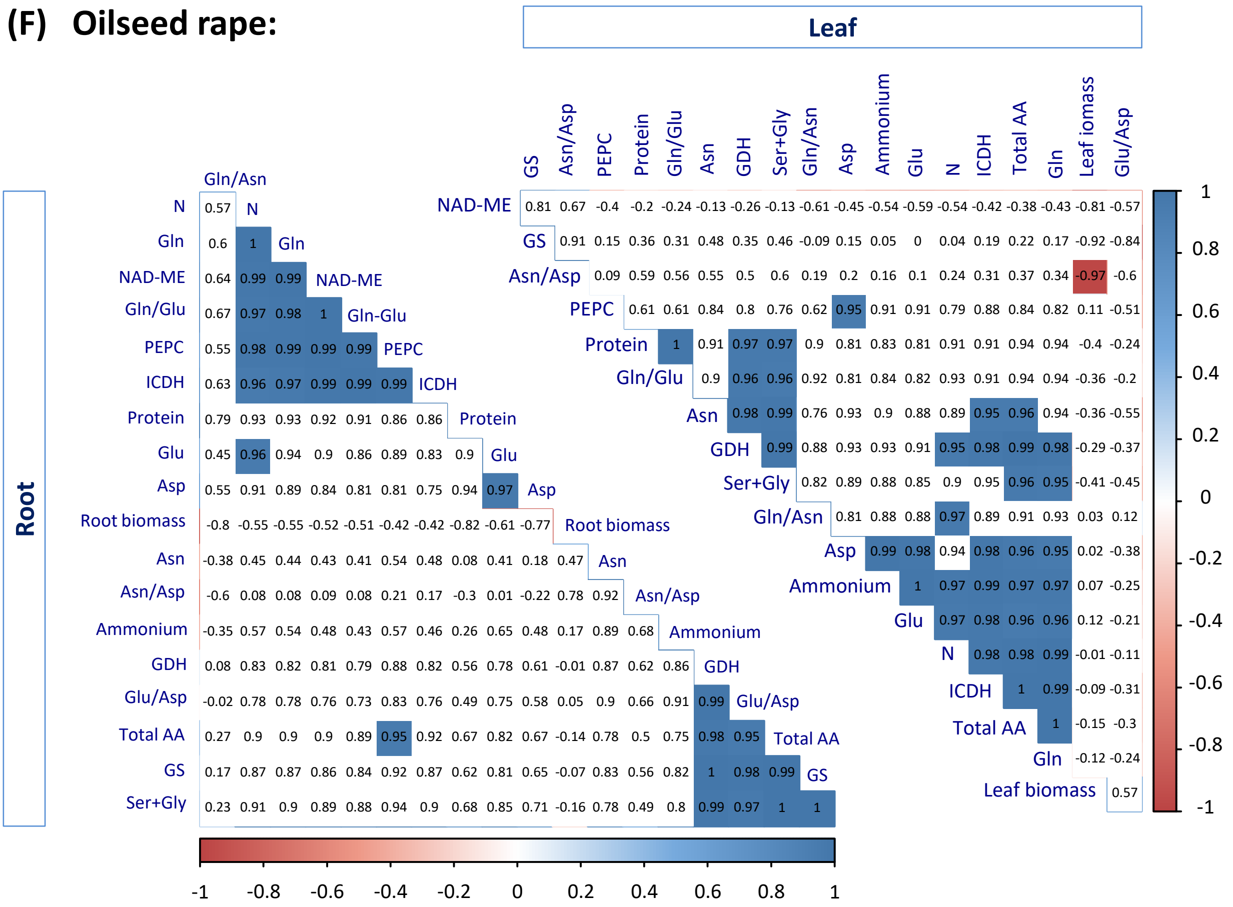

**(G) *Arabidopsis*:**

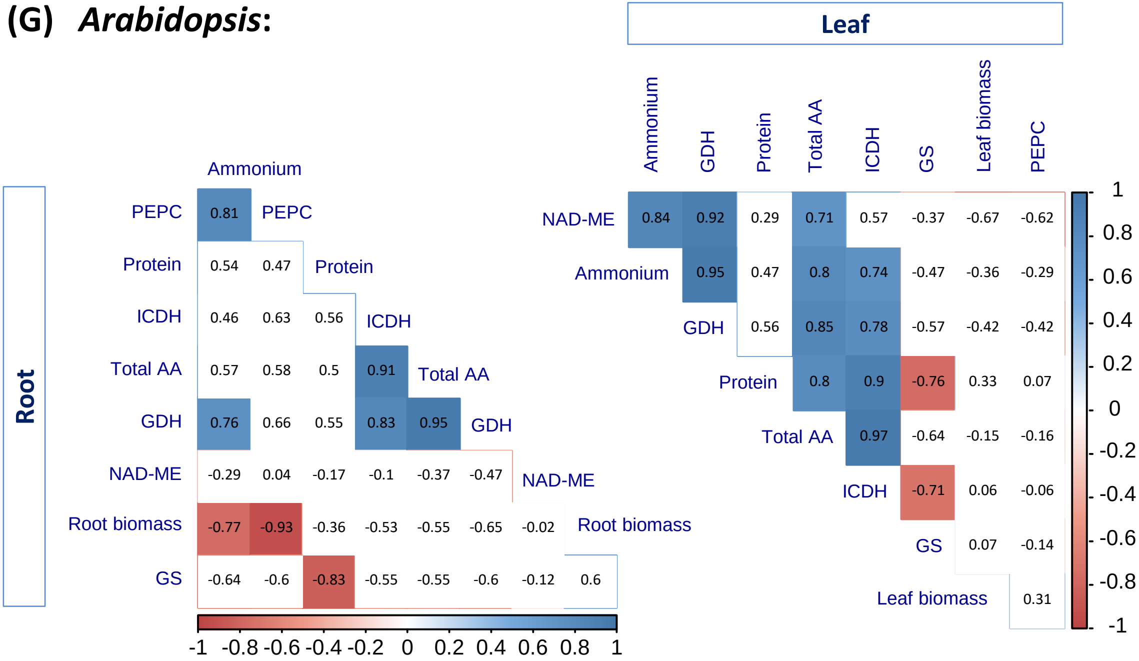

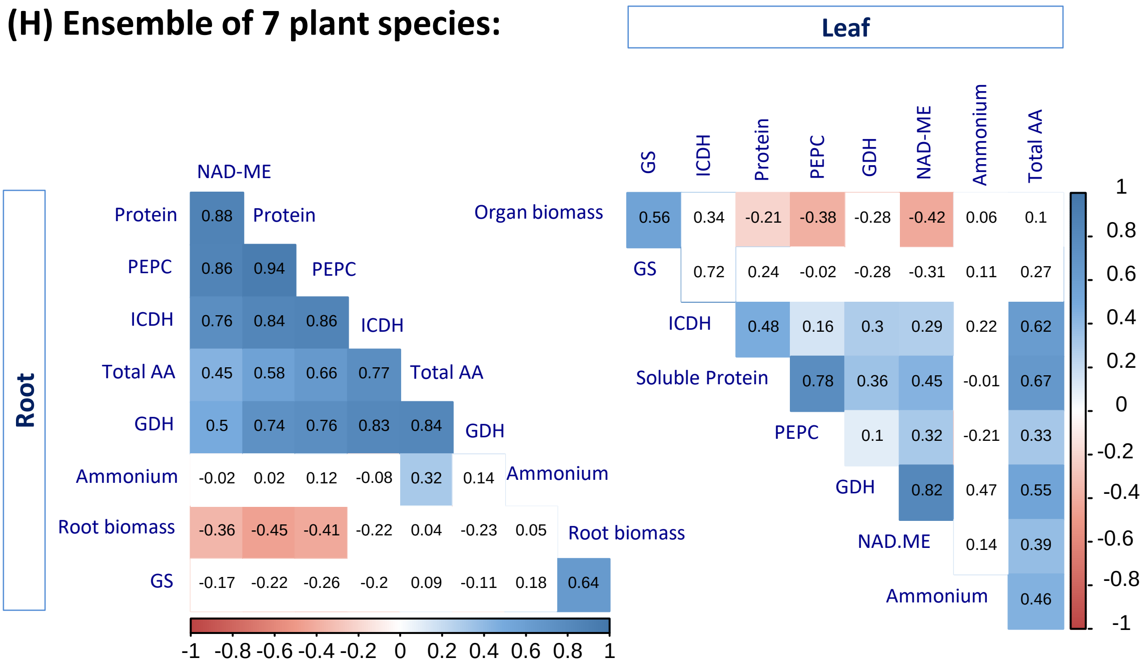
(H) Ensemble of 7 plant species:
Leaf
Root
NAD-ME
Protein
PEPC
ICDH
Total AA
GDH
Ammonium
Root biomass
GS
0.88
0.86 0.94
0.76 0.84 0.86
0.45 0.58 0.66 0.77
0.5 0.74 0.76 0.83 0.84
-0.02 0.02 0.12 -0.08 0.32 0.14
-0.36 -0.45 -0.41 -0.22 0.04 -0.23 0.05
-0.17 -0.22 -0.26 -0.2 0.09 -0.11 0.18 0.64
-1 -0.8 -0.6 -0.4 -0.2 0 0.2 0.4 0.6 0.8 1
GS
ICDH
Protein
PEPC
GDH
NAD-ME
Ammonium
Total AA
Organ biomass
0.56 0.34 -0.21 -0.38 -0.28 -0.42 0.06 0.1
GS
0.72 0.24 -0.02 -0.28 -0.31 0.11 0.27
ICDH
0.48 0.16 0.3 0.29 0.22 0.62
Soluble Protein
0.78 0.36 0.45 -0.01 0.67
PEPC
0.1 0.32 -0.21 0.33
GDH
0.82 0.47 0.55
NAD.ME
0.14 0.39
Ammonium
0.46
1 0.8 0.6 0.4 0.2 0 -0.2 -0.4 -0.6 -0.8 -1
